# A miR858 variant negatively regulates resistance to tea leaf spot through targeting the CsMYB1–*CsPME41* module

**DOI:** 10.1186/s43897-026-00238-7

**Published:** 2026-07-07

**Authors:** Dongqin Zhang, Bin Wang, Zeqi Qi, Jun Zhang, Yu Huang, Delu Wang, Baoan Song, Zhuo Chen

**Affiliations:** 1 State Key Laboratory of Green Pesticides, Guizhou University, Guiyang, China; 2 College of Forestry, Guizhou University, Guiyang, China

**Keywords:** *Tea leaf spot*, *Epicoccum sorghinum*, *Disease resistance*, *Csi-miR858-3p_L-1*, *CsMYB1*, *CsPME41*

## Abstract

In recent years, tea leaf spot, caused by the fungus *Epicoccum sorghinum*, adversely affects both the yield and quality of tea in China. Transient overexpression and antisense oligonucleotide (AsODN) assays revealed that upregulating *CsMYB1*, a transcription factor located in the nucleus, enhances resistance to *E. sorghinum* infection in tea leaves. Overexpression of *CsMYB1* in transgenic *Nicotiana benthamiana* conferred resistance to *Botrytis cinerea*. Multi-omics assays of transiently overexpressed *CsMYB1* in tea leaves indicated that CsMYB1 induces the expression of numerous disease resistance genes. DNA affinity purification sequencing assay indicated that CsMYB1 can activate the expression of the disease resistance gene *pectinesterase*/*pectinesterase inhibitor 41* (*CsPME41*).* β*-glucuronidase and dual-luciferase assays showed that csi-miR858-3p_L-1 targets and cleaves *CsMYB1*. Moreover, transient overexpression and AsODN assays in tea leaves and transgenic csi-miR858-3p_L-1 *N. benthamiana* plants indicated that increasing csi-miR858-3p_L-1 levels heightens susceptibility to *E. sorghinum* in tea leaves. The relative expression levels of *CsMYB1*, *CsPME41*, and csi-miR858-3p_L-1 in tea leaves exhibited distinct spatial and temporal patterns in response to *E. sorghinum* invasion. This study reveals that the csi-miR858-3p_L-1–*CsMYB1*–*CsPME41* module plays a role in the disease resistance response of tea plants to *E. sorghinum* infection, providing crucial data for resistance breeding.

**Supplementary Information:**

The online version contains supplementary material available at 10.1186/s43897-026-00238-7.

## Core

This study demonstrated that the “csi-miR858-3p_L-1–*CsMYB1*–*CsPME41*” module plays a regulatory role in tea plant resistance to *Epicoccum sorghinum*, with both positive and negative contributions. These findings provided critical data to inform resistance breeding strategies aimed at improving tea plant defense against *E. sorghinum*.

## Gene & accession numbers

The raw RNA-seq and small RNA-seq data generated in this study have been deposited in the NCBI SRA under the following BioProject accession numbers: PRJNA799860 (mRNA-seq from *C. sinensis* leaves infected with *E. sorghinum*), PRJNA824005 (small RNA-seq from *C. sinensis* leaves infected with *E. sorghinum*), and PRJNA1248450 (mRNA-seq from *C. sinensis* leaves transiently overexpressing *CsMYB1*).

## Introduction

Tea plant (*Camellia sinensis* (L.) O. Kuntze) is an economically important woody perennial plant, which is widely cultivated in tropical and subtropical regions (Farag et al. 2023; Huang et al. 2022b). Tea leaf spot, caused by the fungus *Epicoccum sorghinum*, is a recently discovered foliar disease in China that significantly negatively affects both the yield and quality of tea leaves (Bao et al. 2019). The impact of diseases on tea yield and quality underscores the need for effective control measures. Currently, research on *E. sorghinum*-induced diseases is limited, and no effective control measures have been established to address this issue (Huang et al. 2022a, 2023a, b). Research on genes affecting tea leaf spot resistance would help us understand the patterns of disease outbreaks and provide valuable accessions for breeding leaf spot-resistant tea cultivars, which would be of use in the prevention and control of the disease.

MYB is an important family of transcription factors (TFs) in plants. Based on the number of repeats of the conserved MYB domain in the N-terminal region (which is involved in DNA binding), plant MYBs are classified into four subfamilies: 1R-MYB (composed of one or two independent repeat sequences), 2R-MYB (R2R3-MYB, composed of two adjacent repeat sequences), 3R-MYB (composed of three adjacent repeats), and 4R-MYB (composed of four adjacent repeats) (Dubos et al. 2010; Wu et al. 2022). Studies have shown that MYB1, a member of the R2R3-MYB family, regulates stress-responsive genes involved in secondary metabolite biosynthesis pathways. As a result, it play a role in plant growth and development, as well as in responses to abiotic stresses (Kanzaki et al. 2020; Legay et al. 2010; Yu et al. 2019; Zhou et al. 2023). For example, MdMYB1 in apple (*Malus* × *domestica*) activated the expression of *glutathione S-transferase 6* (*MdGSTF6*) gene, regulating the biosynthesis and transport of anthocyanins (Jiang et al. 2019). Additionally, GhMYB1a in *Gerbera* × *hybrida* regulated the biosynthesis of flavonols and anthocyanins by activating the expression of *chalcone synthase* (*GhCHS*) and *dihydroflavonol reductase* (*GhDFR*) genes (Zhong et al. 2020). The overexpression of *EgMYB1* from *Eucalyptus gunnii* in *Arabidopsis thaliana* and *Populus* suppressed lignin deposition and cellulose biosynthesis by repressing key genes associated with secondary cell wall formation, highlighting the potential of this gene to reduce recalcitrance in lignocellulosic biomass (Legay et al. 2010). MYB1 in *Chenopodium glaucum* upregulated stress-responsive genes in *Arabidopsis*, such as *superoxide dismutase* and *peroxidase*, thereby enhancing membrane stability under saline and drought conditions (Zhou et al. 2023). CsMYB1 activated the expression of *GLABRA3* and *anthocyanidin reductase* genes, which are involved in trichome formation and regulate the biosynthesis of galloylated catechins in tea plant populations (Li et al. 2022a). However, the molecular mechanism by which CsMYB1 induces disease-resistance responses in tea plants remains unclear.

MicroRNAs (miRNAs) are non-coding small RNA molecules, approximately 20–24 nucleotides in length, processed from primary transcripts by RNA polymerase II and Dicer-like enzymes (Bartel 2004; Ha et al. 2014). They regulated gene expression by interacting with target mRNAs in a sequence-specific manner, resulting in mRNA cleavage or translational repression (Llave et al. 2002; Pasquinelli 2012). miR858 was one of the conserved miRNA families in plants, playing a role in plant growth and development as well as responses to biotic and abiotic stresses (Lin et al. 2021; Piya et al. 2017; Wang et al. 2023). miR858 cleaved and suppressed *AaMYBC1* mRNA, a gene that promotes anthocyanin biosynthesis in kiwifruit (*Actinidia arguta*) (Li et al. 2019). The MdBBX22–miR858–*MdMYB9*/*11*/*12* modules in apple (*M.* × *domestica*) regulated the biosynthesis of proanthocyanidins in apple peel (Zhang et al. 2022), while miR858 in baikal skullcap (*Scutellaria baicalensis*) cleaved and suppressed *SbMYB47* mRNA, SbMYB47 was closely associated with flavonoid biosynthesis (Yang et al. 2024). miR828 and miR858 in cotton (*Gossypium hirsutum*) targeted and cleaved *GhMYB2* mRNA, thereby regulating the development of trichomes, which are involved in the formation of cotton fibers (Guan et al. 2014). Furthermore, miR858 in *Ammopiptanthus mongolicus* was significantly downregulated under drought stress, and the miR858–*MYB* pair enhanced drought stress tolerance (Gao et al. 2016). The overexpression of miR858 cleaved and suppressed the expression of *AtMYB83* mRNA, thereby interfering with the parasitism of *Heterodera schachtii* in *Arabidopsis* (Piya et al. 2017). Transgenic plants expressing miR858 target mimics effectively blocked miR858 activity, leading to the upregulation of its target gene expression, including genes encoding flavonoid-specific TFs *AtMYB11*, *AtMYB12*, and *AtMYB111*, as well as upstream genes in the flavonoid branch of the phenylpropanoid pathway, such as *phenylalanine ammonia lyase 4*, *cinnamate 4-hydroxylase*, and *4-coumarate: CoA ligase*. This upregulation enhances *Arabidopsis* resistance to infections by *Plectosphaerella cucumerina*, *Fusarium oxysporum*, and *Colletotrichum higginsianum* (Camargo-Ramírez et al. 2018).

In a previous study, we sequenced miRNAs and mRNAs from *C. sinensis* leaves inoculated with *E. sorghinum* hyphae (Huang et al. 2022a, 2023a, b). Upon analysis of the mRNAs targeted by the infection-induced miRNAs, the csi-miR858-3p_L-1–*CsMYB1* module was of particular interest due to its potential role in pathogen–host interactions. However, to date, the role of csi-miR858-3p_L-1 in tea plants remains unclear. In the current study, we hypothesize that csi-miR858-3p_L-1–*CsMYB1* pair could play an important role in resistance to tea leaf spot. To test this hypothesis, we analyzed the expression changes of csi-miR858-3p_L-1 and *CsMYB1* during the occurrence of tea leaf spot. The disease-resistance functions of csi-miR858-3p_L-1 and *CsMYB1* were further investigated. *Pectinesterase*/*pectinesterase inhibitor 41* (*CsPME41*), a downstream regulatory gene of CsMYB1, was found to be involved in disease resistance in tea plants. In this study, we demonstrated that the csi-miR858-3p_L-1–*CsMYB1*–*CsPME41* module plays a role in tea plant resistance to *E. sorghinum*. These findings will help clarify the epidemiological patterns of the disease and provide an important foundation for disease control and resistance breeding.

## Results

### CsMYB1 functions as a TF

**Fig. 1:**
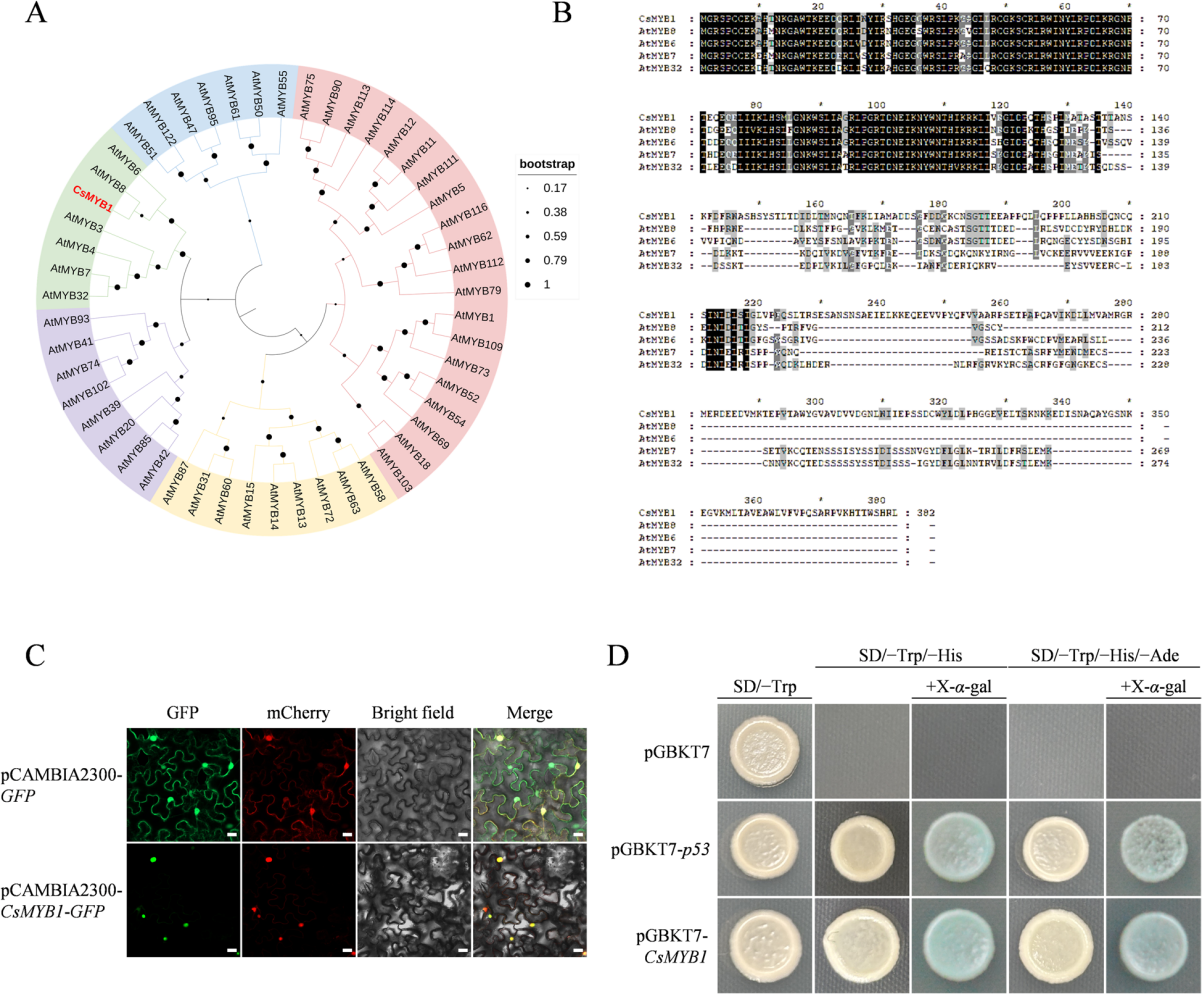
Characterization of *CsMYB1*. **A** Phylogenetic analysis of CsMYB1 (highlighted in red) and other MYB proteins from *Arabidopsis*. **B** Protein sequence alignment of CsMYB1 with *Arabidopsis* MYB (AtMYB8, AtMYB6, AtMYB7, and AtMYB32). **C** Subcellular localization of the pCAMBIA2300-*CsMYB1*-*GFP* fusion protein in *N. benthamiana* epidermal leaf cells. The empty pCAMBIA2300-*GFP* vector was used as a negative control. Scale bars = 20 μm. **D** Transcriptional activation assay of CsMYB1 in yeast cells. Yeast transformants were cultured on SD media: SD/− Trp, SD/− Trp − His, SD/− Trp − His + X-*α*-gal, SD/− Trp − His − Ade, and SD/− Trp − His − Ade + X-*α*-gal. The pGBKT7 empty vector served as the negative control, while yeast cells carrying the pGBKT7-*p53* construct were used as the positive control

Phylogenetic analysis reveals that CsMYB1 was closely related to *Arabidopsis* AtMYB8 and AtMYB6, together forming a distinct clade (Fig. 1A). Protein sequence alignment shows a high degree of homology between CsMYB1 and both AtMYB8 and AtMYB6 (Fig. 1B). Previous studies demonstrated that AtMYB8 and AtMYB6 play regulatory roles in stress responses, flavonoid biosynthesis, and host disease resistance (Gong et al. 2024; Wang et al. 2019). Subcellular localization assay shows that the fluorescence signal of the pCAMBIA2300-*CsMYB1*-*GFP* construct was localized in the nucleus, while the control, pCAMBIA2300-*GFP*, showed fluorescence distributed throughout the entire cell (Fig. 1C). Transcriptional activation assay reveals that colonies expressing pGBKT7-*CsMYB1* grew on synthetic defined (SD) media SD/− Trp/− His supplemented with X-*α*-gal, as well as on SD/− Trp/− His/− Ade media with X-*α*-gal. These colonies developed a blue coloration, similar to that observed for the positive control pGBKT7-*p53*. In contrast, colonies transformed with the negative control construct pGBKT7 does not grow on any of the above SD media (Fig. 1D).

### CsMYB1 positively regulates disease resistance in tea plants

**Fig. 2:**
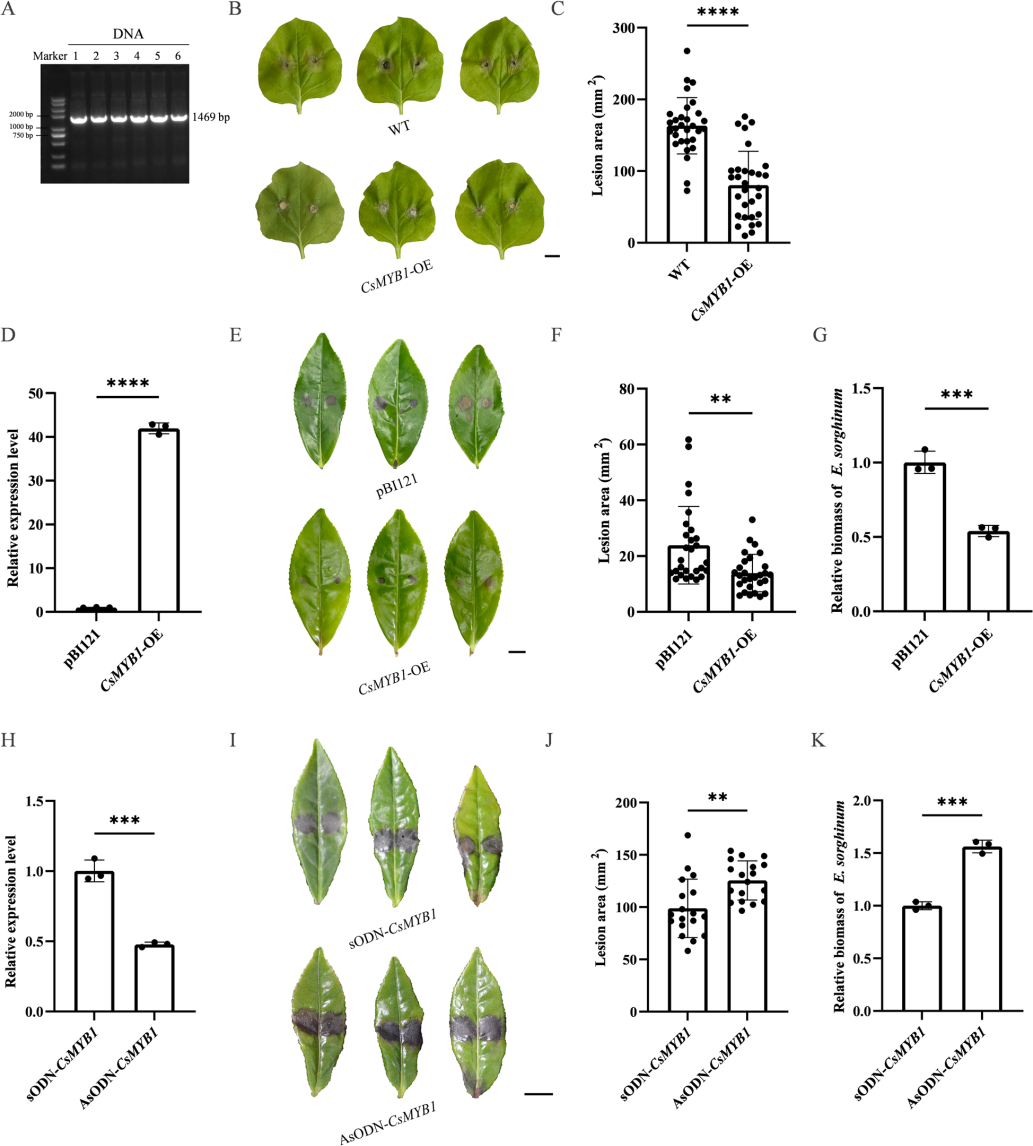
Functional analysis of CsMYB1 in disease resistance. **A** *CsMYB1*-OE (over-expressed) transgenic *N. benthamiana* leaves were verified by PCR using specific primers. **B** Virulence of *B. cinere*a hyphae on *CsMYB1*-OE transgenic *N. benthamiana* leaves, with wild-type *N. benthamiana* as a control, was assessed based on lesion size at 40 hpi. **C** The lesion areas on wild-type *N. benthamiana* leaves and *CsMYB1*-OE transgenic *N. benthamiana* leaves were observed at 40 hpi with *B. cinerea* hyphae (*n* = 30). **D** Relative expression level of *CsMYB1* in tea leaves treated with pBI121 empty vector and *CsMYB1*-OE was analyzed using RT-qPCR assay (*n* = 3). **E** The disease symptoms of *CsMYB1*-OE-transfected tea leaves and pBI121 control at 48 hpi with *E. sorghinum* hyphae. **F** The lesion areas on *CsMYB1*-OE-transfected tea leaves and pBI121 control were observed at 48 hpi with *E. sorghinum* hyphae (*n* = 28). **G** DNA content of *E. sorghinum* hyphae in pBI121 control and *CsMYB1*-OE tea leaves at 48 hpi with the hyphae (*n* = 3). **H** Relative expression level of *CsMYB1* in AsODN-treated and sODN-treated leaves were analyzed using RT-qPCR assay (*n* = 3). **I** The disease symptoms of AsODN-*CsMYB1*-treated and sODN-*CsMYB1*-treated tea leaves at 48 hpi with *E. sorghinum* hyphae. **J** The lesion areas on AsODN-*CsMYB1*-treated and sODN-*CsMYB1*-treated leaves were observed at 48 hpi with *E. sorghinum* hyphae (*n* = 18). **K** DNA content of *E. sorghinum* hyphae in AsODN-*CsMYB1*-treated and sODN-*CsMYB1*-treated leaves at 48 hpi with the hyphae (*n* = 3). All scale bars = 1 cm. Data are presented as mean ± standard deviation. Statistical significance was determined by Student’s *t*-test (**P < 0.01, ***P < 0.001, ****P < 0.0001)

To investigate the potential role of CsMYB1 in disease resistance in tea leaves inoculated with *E. sorghinum* hyphae, studies were conducted on transgenic *Nicotiana benthamiana* lines overexpressing *CsMYB1*, transient *CsMYB1*-overexpressed tea leaves, and antisense oligonucleotide (AsODN) silencing assays in tea leaves. PCR assay confirms the stable overexpression of *CsMYB1* in *N. benthamiana* plants, indicating that *CsMYB1*-overexpressed transgenic plants were successfully generated (Fig. 2A). At 40 h post-inoculation (hpi) with *Botrytis cinerea*, the transgenic tobacco plants exhibits smaller lesion areas than the control plants (Fig. 2B, C). Reverse-transcription quantitative PCR (RT-qPCR) assay shows a 42-fold upregulation of *CsMYB1* expression in *CsMYB1*-OE treatment compared to the pBI121 empty vector control (Fig. 2D). At 48 hpi with *E. sorghinum*, the lesion area is significantly smaller in the treated leaves than in the control leaves (Fig. 2E, F), and the fungal DNA content in the *CsMYB1*-OE treatment is significantly lower than that in the pBI121 treatment (Fig. 2G). After *CsMYB1* mRNA was transiently silenced in tea leaves using AsODN for 48 h, the relative expression level in the AsODN-*CsMYB1*-treated leaves is significantly lower than that in the control leaves treated with sense oligonucleotide (sODN-*CsMYB1*) (Fig. 2H). Tea leaves treated with AsODN exhibit significantly larger lesion areas than those treated with sODN at 48 hpi with *E. sorghinum* (Fig. 2I, J), and the fungal DNA content in the AsODN-*CsMYB1* treatment is significantly higher than that in the sODN-*CsMYB1* treatment (Fig. 2K). Based on the results of these assays, we concluded that CsMYB1 plays a role in disease resistance to *E. sorghinum* hyphae.

### Transcriptomic and metabolomic analysis to identify genes and metabolites affected by CsMYB1

**Fig. 3:**
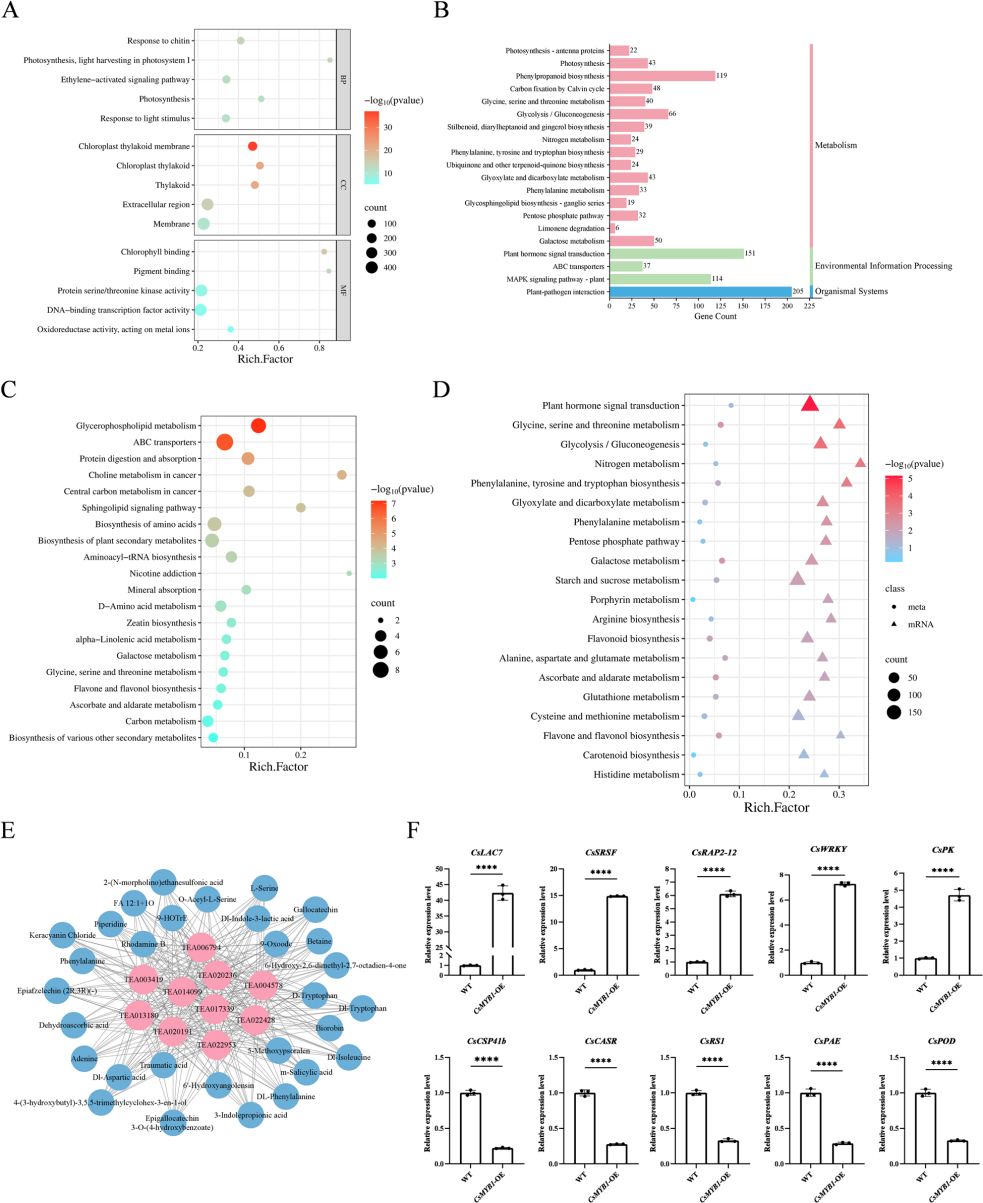
Integrated transcriptomic and metabolomic analysis of *CsMYB1* transient overexpression in tea leaves. **A** GO enrichment analysis of DEGs from transcriptomic data. The top five enriched terms in biological processes, molecular functions, and cellular components are displayed. **B** KEGG enrichment of DEGs. The top 20 enriched pathways are shown. **C** The top 20 pathways in KEGG enrichment analysis of DAMs from metabolomic data. **D** Bubble plot of co-enriched KEGG pathways shared by DEGs and DAMs. **E** Interaction network between ten stress-related DEGs (pink nodes) and highly correlated DAMs (blue nodes). **F** Relative expression levels of the ten DEGs identified in the transcriptome were validated uisng RT-qPCR assay. Data are presented as mean ± standard deviation. Statistical significance was determined by Student’s *t*-test (****P < 0.0001)

To better understand the molecular mechanisms regulated by *CsMYB1*, tea leaves were subjected to transient *CsMYB1*-overexpression (*CsMYB1-*OE) for 48 h, followed by transcriptomic, metabolomic, and bioinformatic analyses. Compared with the wild-type (WT) treatment, the *CsMYB1*-OE plants induce a total of 5,560 differentially expressed genes (DEGs), namely 2,720 upregulated genes and 2,840 downregulated genes (Supplemental Fig. S1; Supplemental Table S1). Gene Ontology (GO) enrichment analysis reveals that the DEGs were primarily involved in terms such as protein serine/threonine kinase activity, DNA-binding transcription factor activity, oxidoreductase activity acting on metal ions, response to chitin, photosynthesis, light harvesting in the photosystem, ethylene-activated signaling pathway, and response to light stimulus, among others (Fig. 3A). The top three enriched Kyoto Encyclopedia of Genes and Genomes (KEGG) terms of the DEGs are plant-pathogen interaction, plant hormone signal transduction, and phenylpropanoid biosynthesis (Fig. 3B). In *CsMYB1*-OE tea leaves, 49 differentially abundant metabolites (DAMs) are significantly increased, and 61 DAMs are significantly decreased (Supplemental Fig. S2; Supplemental Table S2). The top four enriched KEGG terms of the DAMs are glycerophospholipid metabolism, ABC transporters, biosynthesis of amino acids, and biosynthesis of plant secondary metabolites (Fig. 3C).

KEGG enrichment analysis of correlated DEGs and DAMs reveals that the most enriched terms were cysteine and methionine metabolism, histidine metabolism, and carotenoid biosynthesis (Fig. 3D; Supplemental Table S3). An integrated analysis of the DEGs and DAMs is performed to construct a gene–metabolite interaction network (Fig. 3E), which identified ten core regulatory genes: *laccase-7 like* (*CsLAC7*), *serine/arginine-rich splicing factor-like* (*CsSRSF*), *ethylene-responsive transcription factor ERF RAP2-12* (*CsRAP2-12*), *CsWRKY*, *pyruvate kinase* (*CsPK*), *chloroplast stem-loop binding protein of 41 kDa b* (*CsCSP41b*), *calcium sensing receptor-like* (*CsCASR*), *raffinose synthase 1* (*CsRS1*), *pectin acetylesterase* (*CsPAE*), and *peroxidase* (*CsPOD*). These DEGs show strong co-expression relationships with various functional metabolites, including phenolic secondary metabolites (gallocatechin, epiafzelechin, *m*-salicylic acid, and epigallocatechin 3-*O*-(4-hydroxybenzoate)), lipid derivatives (FA 12:1 + 1O, 9-HOTrE, and traumatic acid), amino acids (DL-isoleucine, L-serine, DL-phenylalanine, and DL-tryptophan), and other bioactive compounds (dehydroascorbic acid, 3-indolepropionic acid, and 6-hydroxy-2,6-dimethyl-2,7-octadien-4-one) (Fig. 3E). RT-qPCR assay confirms the upregulation of *CsLAC7*, *CsSRSF*, *CsRAP2-12*, *CsWRKY*, and *CsPK*, and the downregulation of *CsCSP41b*, *CsCASR*, *CsRS1*, *CsPAE*, and *CsPOD*, with trends that were highly consistent with the transcriptomic data (Fig. 3F; Supplemental Table S4). These results confirmed the strong positive correlation and reliability between the transcriptomic and metabolomic data. Based on the transcriptomic and metabolomic data from tea leaves transiently overexpressing *CsMYB1*, we found that CsMYB1 plays a role in enhancing plant defense responses in tea leaves.

### DNA affinity purification sequencing (DAP-seq) on CsMYB1

**Fig. 4:**
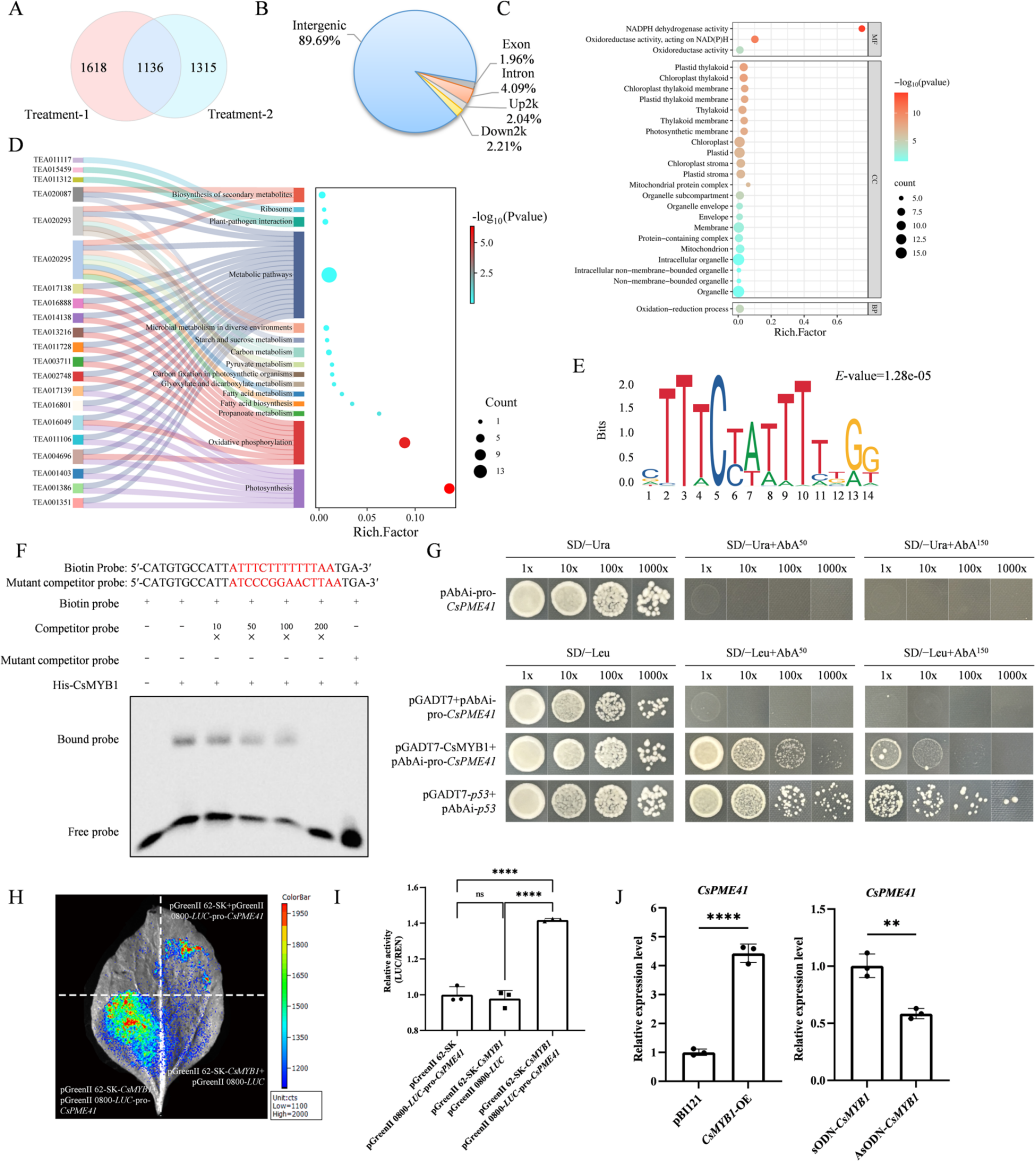
DAP-seq data analysis of CsMYB1 and validation of the interaction between the CsMYB1–*CsPME41* pair. **A** Venn diagram illustrating the overlap of CsMYB1-DNA binding peaks between two technical replicates. **B** Genome-wide distribution of CsMYB1-DNA binding peaks across functional genomic regions (e.g., promoters, exons, and introns). **C** GO enrichment analysis of genes associated with CsMYB1-bound peaks. **D** KEGG pathway Sankey diagram integrated with bubble plot visualization, depicting enriched pathways linked to CsMYB1-binding genes. Bubble size corresponds to the number of genes per pathway. **E** Predicted binding motif of CsMYB1 within the *CsPME41* promoter region. **F** EMSA analysing the binding of the fusion protein His-CsMYB1 to the *CsPME41* promoters in vitro.. Unlabeled probes at 200-fold, 100-fold, 50-fold, and 10- fold excess of the biotin-labelled probe were used as competitors. Symbols: ‘ + ’, presence; ‘ − ’, absence. **G** Y1H analysis of CsMYB1 binding to the *CsPME41* promoter (pro-*CsPME41*). The pAbAi-pro-*CsPME41* construct was tested for auto-activation on selective SD/− Ura medium containing 0, 50, or 150 ng/mL aureobasidin A (AbA). Y1H yeast cells co-transformed with pGADT7 empty vector + pAbAi-pro-*CsPME41*, pGADT7-*p53* + pAbAi-*p53*, or pGADT7-*CsMYB1* + pAbAi-pro-*CsPME41* were cultured on SD/− Leu medium. Growth was assessed on selective SD/− Leu medium supplemented with 0, 50, or 150 ng/mL AbA. The pGADT7 empty vector + pAbAi-pro-*CsPME41* and pGADT7-*p53* + pAbAi-*p53* served as control. **H** Schematic diagram of Dual-LUC reporter assay. The effector-reporter combination pGreenII 62-SK-*CsMYB1* + pGreenII 0800-*LUC*-pro-*CsPME41* was co-infiltrated into *N. benthamiana* leaves. Control infiltrations included pGreenII 62-SK empty vector + pGreenII 0800-*LUC*-pro-*CsPME41* and pGreenII 62-SK-*CsMYB1* + pGreenII 0800-*LUC* empty vector. **I** Firefly luciferase and *Renilla* luciferase activity quantification using a Dual-Luciferase Reporter Gene Assay Kit. **J** Relative expression levels of *CsPME41* in tea leaves treated with transient overexpression or transient silencing in OE or AsODN assays were analyzed using RT-qPCR assay. Data are presented as mean ± standard deviation. Statistical significance was determined by Student’s *t*-test (****P < 0.0001, ns *P* > 0.05)

DAP-seq assay was performed to identify the target genes of CsMYB1. In the Treatment-1 and Treatment-2 replicates, 2,754 and 2,451 peaks are observed, respectively, with 1,136 common peaks (Fig. 4A). The distribution of CsMYB1-binding sites within annotated gene regions was then analyzed. It is found that 2.04% of the sites were enriched in the gene promoter region (2 kb upstream of the transcription start site, TSS), 89.69% in intergenic regions, 1.96% in exonic regions, and 4.09% in intronic regions (Fig. 4B). Next, the frequency distribution of CsMYB1-binding sites within the 10 kb region upstream and downstream of the TSS is determined (Supplemental Fig. S3). CsMYB1 is found to preferentially bind to DNA sequences near the TSS, with a high enrichment within the first 1,000 bp (Supplemental Fig. S4). GO enrichment analysis of the genes where the promoters bound to CsMYB1 reveals that CsMYB1 regulates genes involved in NADPH dehydrogenase activity, oxidoreductase activity, acting on NAD(P)H, chloroplasts, plastids, plastid thylakoids, and chloroplast thylakoids, among others (Fig. 4C). According to KEGG analysis, these genes are enriched in terms related to photosynthesis and oxidative phosphorylation (Fig. 4D). Based on the enrichment analysis of the GO and KEGG databases, we further screen *CsPME41* as a target gene of CsMYB1 and predict the binding motif of the *CsPME41* promoter to CsMYB1 (Fig. 4E; Supplemental Table S5).

### CsMYB1 activates the expression of CsPME41 gene

Electrophoretic mobility shift assay (EMSA), yeast one-hybrid (Y1H), and dual-luciferase (Dual-LUC) reporter assays were used to investigate whether CsMYB1 binds to the *CsPME41* promoter and induces its expression. EMSA result demonstrates that incubation of CsMYB1 with a biotin-labeled *CsPME41* probe resulted in a specific binding band. The intensity of the shifted band gradually decreased with increasing concentrations of the competitor probe, whereas the mutated competitor probe completely abolished the binding effect. These findings indicate that CsMYB1 directly binds to the *CsPME41* promoter (Fig. 4F). The result shows that Y1H Gold Yeast co-transfected with pGADT7-*CsMYB1* and pAbAi-pro-*CsPME41* could grow, indicating that CsMYB1 can bind to the *CsPME41* promoter and activate the expression of *CsPME41* (Fig. 4G). Dual-LUC assay reveals that the fluorescence intensity in the region of the leaf co-injected with pGreenII 62-SK-*CsMYB1* and pGreenII 0800-*LUC*-pro-*CsPME41* was significantly higher than that in the control groups, pGreenII 62-SK + pGreenII 0800-*LUC*-pro*-CsPME41* or pGreenII 62-SK-*CsMYB1* + pGreenII 0800-*LUC* (Fig. 4H). The enzyme activity assay also shows a significant increase in the Firefly/*Renilla* luciferase (LUC/REN) luminescence ratio in the co-injection group of pGreenII 62-SK-*CsMYB1* and pGreenII 0800-pro-*CsPME41* (Fig. 4I). RT-qPCR assay indicates that the relative expression level of *CsPME41* in the *CsMYB1*-OE treatment were 3.4-fold higher compared to the pBI121 control treatment, while the relative expression level of *CsPME41* in the AsODN-*CsMYB1* treatment were reduced by 41.7% compared to the sODN-*CsMYB1* treatment (Fig. 4J). These results suggested that CsMYB1 acts as a transcriptional activator of *CsPME41* gene.

### CsPME41 positively regulates disease resistance in tea plants

**Fig. 5:**
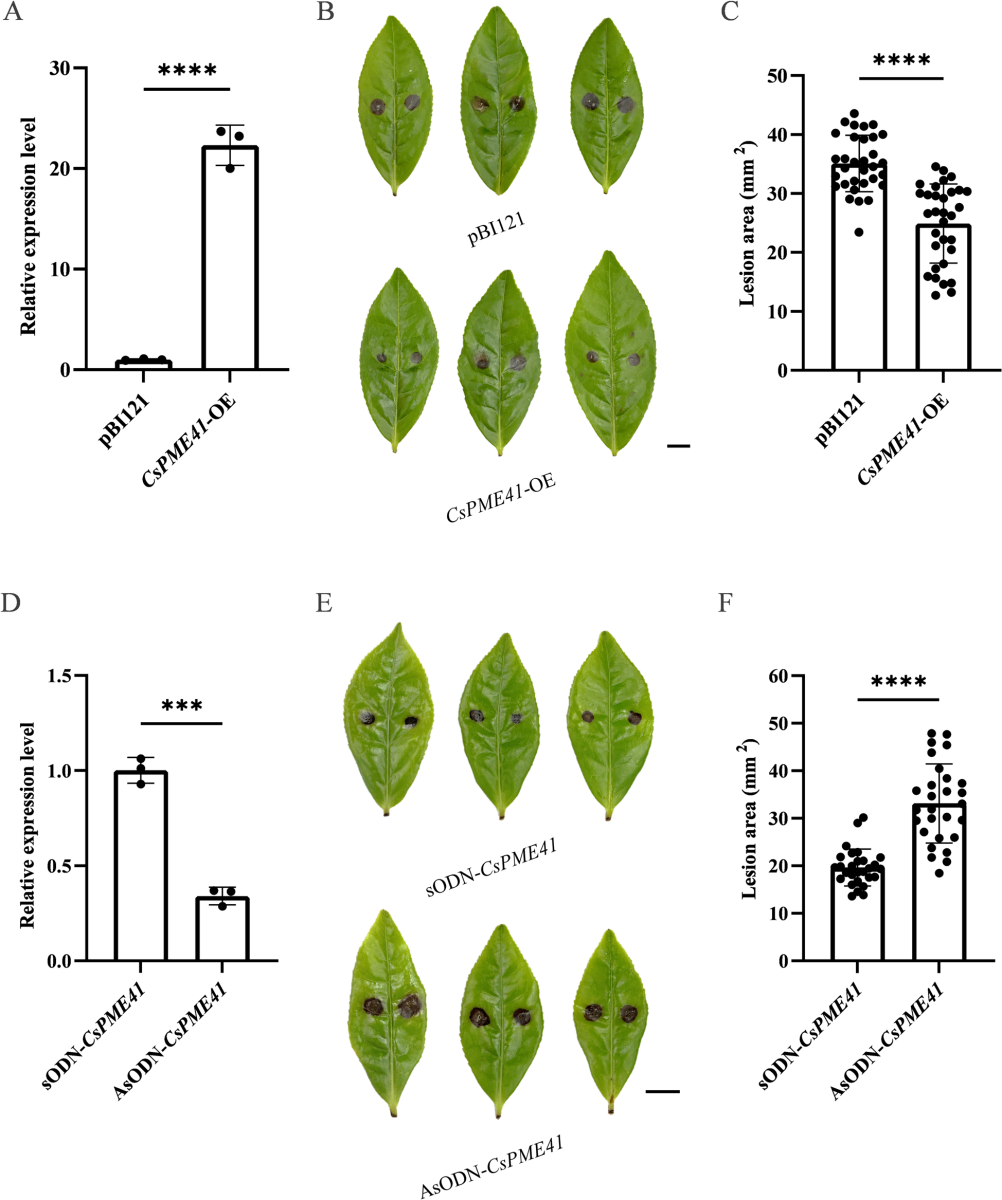
Functional analysis of CsPME41 in disease resistance. **A** Relative expression level of *CsPME41* in tea leaves treated with pBI121 empty vector and *CsPME41*-OE was analyzed using RT-qPCR assay (*n* = 3). **B** The disease symptoms of *CsPME41-*OE-transfected tea leaves and pBI121 control at 48 hpi with *E. sorghinum* hyphae. **C** The lesion areas on *CsPME41*-OE-transfected tea leaves and pBI121 control were observed at 48 hpi with *E. sorghinum* hyphae (*n* = 28). **D** Relative expression level of *CsPME41* in tea leaves treated with AsODN-*CsPME41* and sODN-*CsPME41* was analyzed using RT-qPCR assay (*n* = 3). **E** The disease symptoms of AsODN-*CsPME41*-treated and sODN-*CsPME41*-treated tea leaves at 48 hpi with *E. sorghinum* hyphae. **F** The lesion areas on AsODN-*CsPME41*-treated and sODN-*CsPME41*-treated leaves were observed at 48 hpi with *E. sorghinum* hyphae (*n* = 28). All scale bars = 1 cm. Data are presented as mean ± standard deviation. Statistical significance was determined by Student’s t-test (***P < 0.001, ****P < 0.0001)

To investigate the potential role of CsPME41 in disease resistance in tea leaves inoculated with *E. sorghinum* hyphae, studies were conducted on transient *CsPME41*-overexpressed tea leaves and AsODN assays in tea leaves. RT-qPCR assay indicates that the relative expression level of *CsPME41* in the *CsPME41*-OE treatment was 21.3-fold higher compared to the pBI121 control treatment (Fig. 5A). At 48 hpi with *E. sorghinum*, the lesion area is significantly smaller in the treated leaves than in the control leaves (Fig. 5B, C). After *CsPME41* mRNA in tea leaves were suppressed using AsODN assay for 48 h, the relative expression level of *CsPME41* in the AsODN-*CsPME41* treatment was reduced by 66.0% compared to the sODN-*CsPME41* treatment (Fig. 5D). Tea leaves treated with AsODN exhibit significantly larger lesion areas than those treated with sODN at 48 hpi with *E. sorghinum* (Fig. 5E, F). Based on the results of these assays, we concluded that CsPME41 plays a role in enhancing disease resistance to *E. sorghinum* hyphae.

### csi-miR858-3p_L-1 can cleave CsMYB1 mRNA

**Fig. 6:**
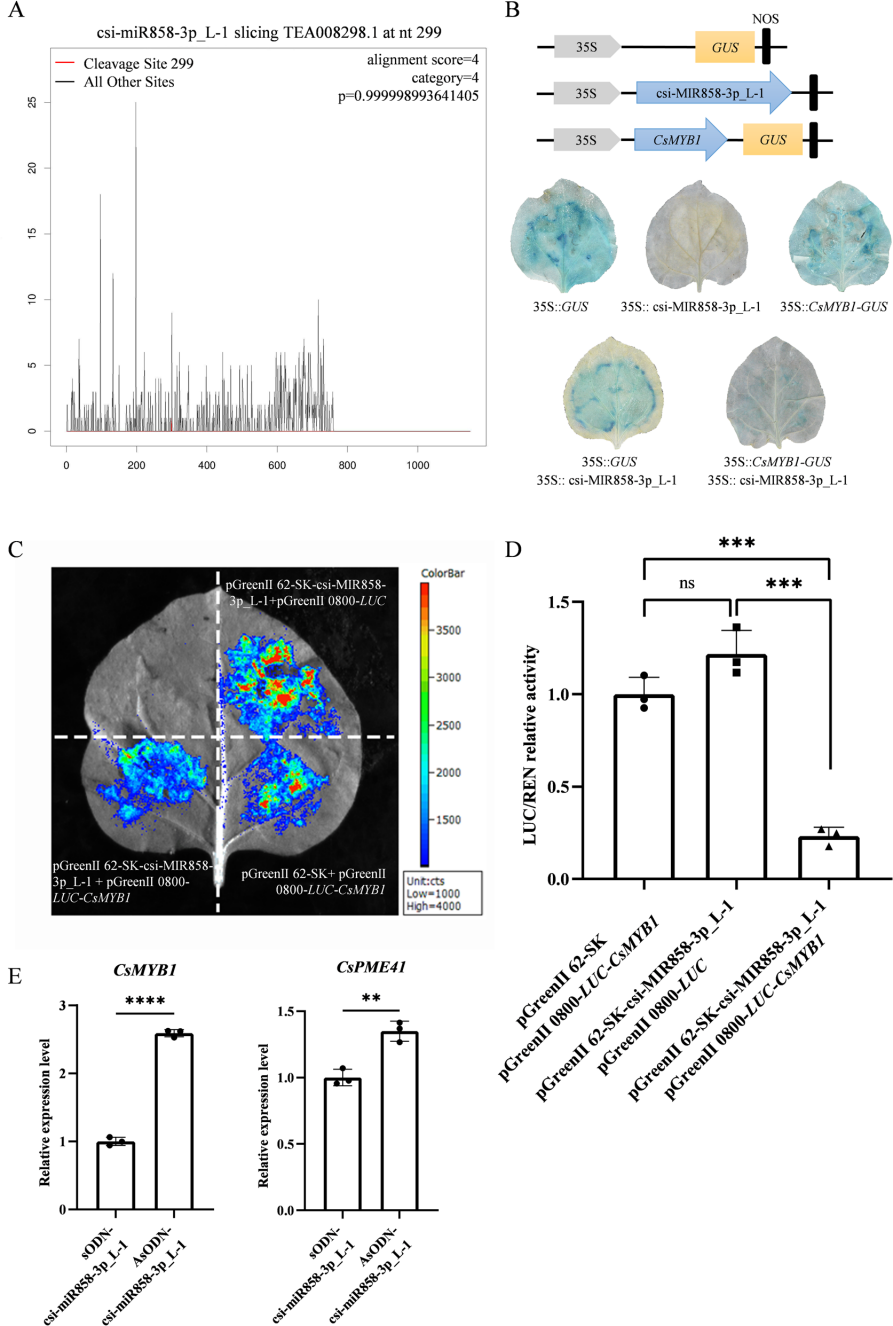
Targeted cleavage of *CsMYB1* mRNA by csi-miR858-3p_L-1. **A** Degradome sequencing identifies csi-miR858-3p_L-1-mediated cleavage of *CsMYB1* mRNA at nucleotide position 299. Red lines denote cleavage sites validated by degradome reads, while black lines represent other potential sites. **B** GUS histochemical staining assay in *N. benthamiana* leaves. Constructs: 35S::*GUS* (positive control), 35S::csi-MIR858-3p_L-1 (negative control), and 35S::csi-MIR858-3p_L-1 + 35S::*CsMYB1*-*GUS* (experimental group). **C** Dual-LUC assay. Co-infiltration of pGreenII 62-SK-csi-MIR858-3p_L-1 + pGreenII 0800-*LUC*-*CsMYB1* and control combinations (pGreenII 62-SK + pGreenII 0800-*LUC*-*CsMYB1* and pGreenII 62-SK-csi-MIR858-3p_L-1 + pGreenII 0800-*LUC*) into tobacco leaves. **D** Firefly luciferase and *Renilla* luciferase activity quantification using a Dual Luciferase Reporter Gene Assay Kit. **E** Relative expression levels of *CsMYB1* and *CsPME41* in tea leaves treated with AsODN-csi-miR858-3p_L-1 and sODN-csi-miR858-3p_L-1 were analyzed using RT-qPCR assay (*n* = 3). Data are presented as mean ± standard deviation. Statistical significance was determined by Student’s *t*-test (***P < 0.001, ns *P* > 0.05)

To verify the cleavage of *CsMYB1* mRNA by csi-miR858-3p_L-1, degradome sequencing is used in the current study to confirm the interaction between csi-miR858-3p_L-1 and *CsMYB1* mRNA and reveals that csi-miR858-3p_L-1 cleaved *CsMYB1* mRNA at nucleotide position 299 (Fig. 6A). To further confirm that csi-miR858-3p_L-1 could cleave *CsMYB1* mRNA *in planta*, histochemical *β*-glucuronidase (GUS) staining reveals that the staining density in the leaves injected with 35S::*GUS*, 35S::*CsMYB1*, and co-injected with 35S::csi-MIR858-3p_L-1 and 35S::*GUS* was significantly deeper due to the expression of *GUS* gene. Nevertheless, the staining density of the area in leaves corresponding to the co-injection site of 35S::csi-MIR858-3p_L-1 and 35S::*CsMYB1* is relatively light due to the cleavage effect of csi-MIR858-3p_L-1 on *CsMYB1* mRNA. In addition, the staining density in the area of leaves injected with 35S::csi-MIR858-3p_L-1 is relatively light, as no GUS expression was detected in the leaves due to the absence of 35S::*GUS* (Fig. 6B). Dual-LUC assay demonstrates a significant decrease in the luminescence value in the region co-injected with pGreenII 62-SK-csi-MIR858-3p_L-1 and pGreenII 0800-*LUC*-*CsMYB1*, compared with the co-injections of pGreenII 62-SK-csi-MIR858-3p_L-1 and pGreenII 0800-*LUC* or pGreenII 62-SK and pGreenII 0800-*LUC*-*CsMYB1* (Fig. 6C). Meanwhile, the LUC/REN ratio of the treatment is also significantly decreased (Fig. 6D). RT-qPCR assay indicates that the relative expression levels of *CsMYB1* and *CsPME41* in AsODN-csi-miR858-3p_L-1 treatment were 1.6- or 0.3-fold higher compared to the sODN-csi-miR858-3p_L-1 treatment (Fig. 6E). All results indicated that csi-miR858-3p_L-1 could directly cleave *CsMYB1* and thereby negatively regulate its expression.

### csi-miR858-3p_L-1 negatively regulates plant disease resistance

**Fig. 7:**
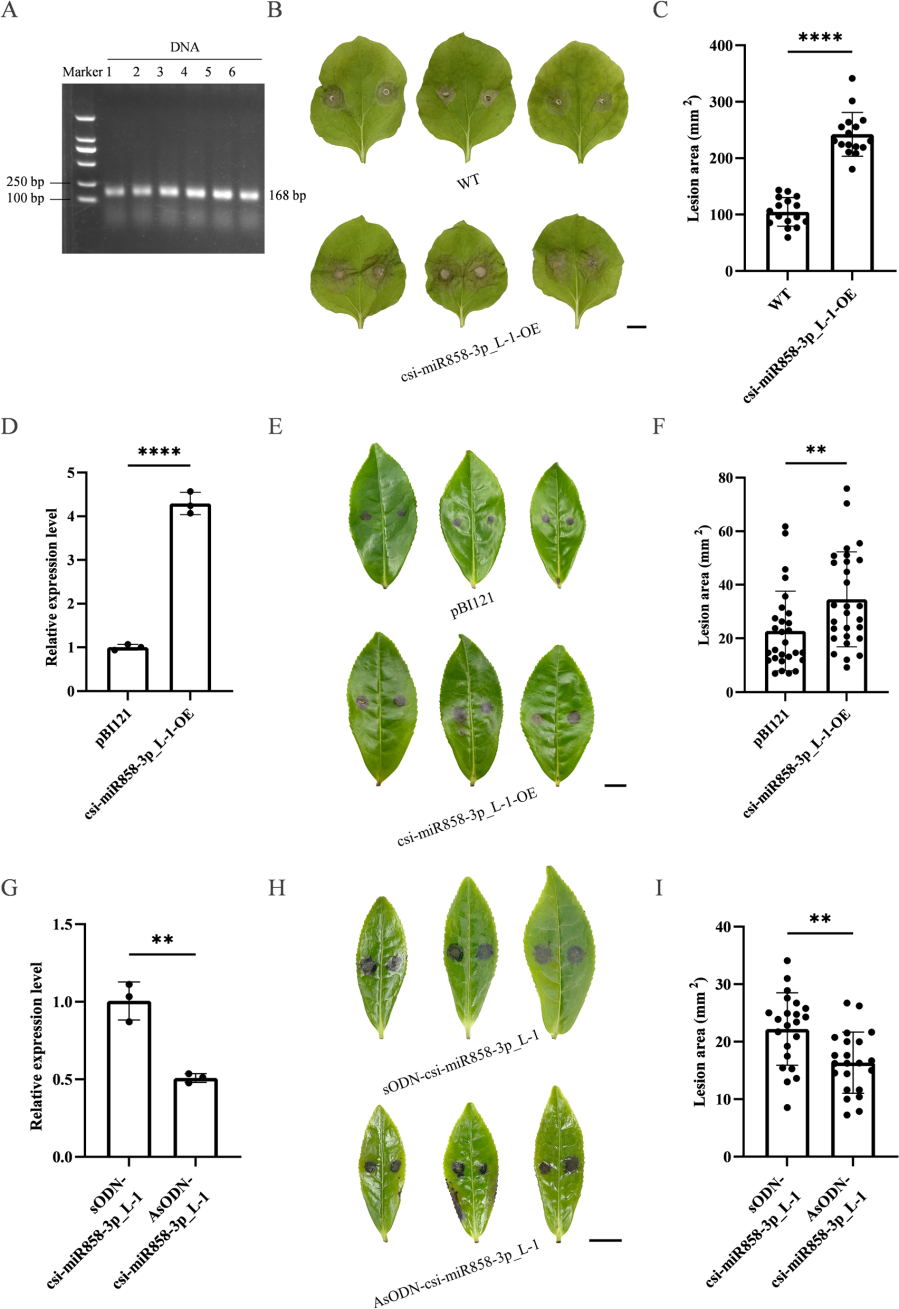
Functional role of csi-miR858-3p_L-1 in disease resistance. **A** csi-miR858-3p_L-1-OE transgenic *N. benthamiana* leaves were verified by PCR using specific primers. **B** Virulence of *B. cinere*a hyphae on *pre-csi-miR858-3p_L-1*-OE transgenic *N. benthamiana* leaves, with wild-type *N. benthamiana* as a control, was assessed based on lesion size at 40 hpi. **C** The lesion areas on wild-type *N. benthamiana* leaves and csi-miR858-3p_L-1-OE transgenic *N. benthamiana* leaves were observed at 40 hpi with *B. cinerea* hyphae (*n* = 16). **D** Relative expression level of csi-miR858-3p_L-1 in tea leaves treated with pBI121 empty vector and OE-csi-miR858-3p_L-1 was analyzed using RT-qPCR assay (*n* = 3). **E** The disease symptoms of csi-miR858-3p_L-1*-*OE-transfected tea leaves and pBI121 control at 48 hpi with *E. sorghinum* hyphae. **F** The lesion areas on csi-miR858-3p_L-1-OE-transfected tea leaves and pBI121 control were observed at 48 hpi with *E. sorghinum* hyphae (*n* = 28). **G** Relative expression level of csi-miR858-3p_L-1 in AsODN-treated and sODN-treated leaves were analyzed using RT-qPCR assay (*n* = 3). **H** The disease symptoms of AsODN-csi-miR858-3p_L-1-treated and sODN-csi-miR858-3p_L-1-treated tea leaves at 48 hpi with *E. sorghinum* hyphae. **I** The lesion areas on AsODN-csi-miR858-3p_L-1-treated and sODN-csi-miR858-3p_L-1-treated leaves were observed at 48 hpi with *E. sorghinum* hyphae (*n* = 22). All scale bars = 1 cm. Data are presented as mean ± standard deviation. Statistical significance was determined by Student’s *t*-test (**P < 0.01, ****P < 0.0001)

To study the role of csi-miR858-3p_L-1 in disease resistance of tea plants, csi-miR858-3p_L-1 was overexpressed in transgenic *N. benthamiana* plants. PCR assay confirms the stable overexpression of csi-miR858-3p_L-1 in *N. benthamiana* plants, indicating that csi-miR858-3p_L-1-OE transgenic plants had been successfully generated (Fig. 7A). When the transgenic *N. benthamiana* leaves were inoculated with *B. cinerea*, the lesion areas in the transgenic plants are found to be significantly larger than those in the control (Fig. 7B, C). In addition, the pBI121-csi-miR858-3p_L-1 vector is infiltrated into tea leaves and RT-qPCR assay indicates that the expression level of csi-miR858-3p_L-1 in the csi-miR858-3p_L-1-OE treatment was significantly higher than that in the empty pBI121 vector treatment (Fig. 7D). Virulence assay reveals that the transiently csi-miR858-3p_L-1-overexpressed tea leaves produced larger lesions areas than the control plants after inoculation with *E. sorghinum* hyphae (Fig. 7E, F). To further verify the effect of csi-miR858-3p_L-1 on disease resistance, AsODN assay was used to downregulate the expression of csi-miR858-3p_L-1 in tea leaves. RT-qPCR assay shows that the expression of csi-miR858-3p_L-1 was significantly silenced by AsODN, reaching 49.7% of the control level (Fig. 7G). After inoculation with *E. sorghinum* hyphae, the lesion areas on tea leaves with silenced miR858-3p expression (via AsODN treatment) are significantly smaller than those on non-silenced leaves treated with sODN treatment (Fig. 7H, I). These results indicated that csi-miR858-3p_L-1 negatively regulates the defense response of tea plants.

### Expression changes of CsMYB1 mRNA, CsPME41 mRNA, and csi-miR858-3p_L-1 in tea leaves in response to infection by E. sorghinum hyphae

**Fig. 8:**
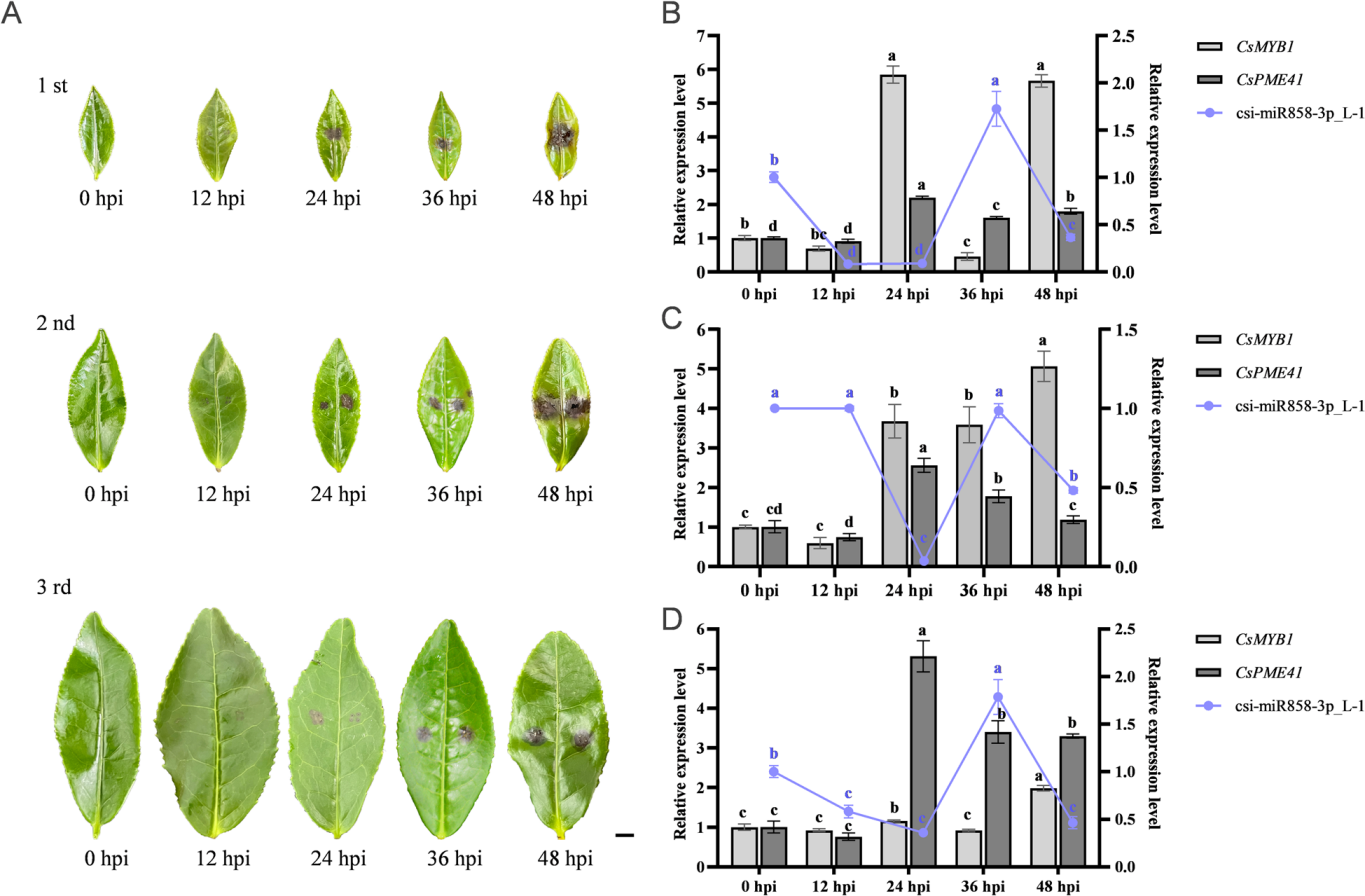
Spatiotemporal expression of *CsMYB1* mRNA, *CsPME41* mRNA, and csi-miR858-3p_L-1 in tea leaves inoculated with *E. sorghinum* hyphae. **A** The detached tea leaves were inoculated with *E. sorghinum* hyphal plugs for 12, 24, 36, and 48 h. ‘1 st’, ‘2 nd’, and ‘3 rd’ represent the first, second, and third tea leaves, respectively. Uninoculated leaves (0 hpi) served as negative controls. All scale bars = 1 cm. **B–D** Relative expression levels of *CsMYB1*, *CsPME41*, and csi-miR858-3p_L-1 in the first (B), second (C), and third (D) tea leaves at various hpi with *E. sorghinum* hyphae. Data are presented as mean ± standard deviation. Statistical significance was determined by analysis of variance and Tukey test (*P* < 0.05); Different letters within a panel signify significant differences (*P* < 0.05)

To determine whether the abundance levels of *CsMYB1* mRNA, *CsPME41* mRNA, and csi-miR858-3p_L-1 in tea leaves respond to infection by *E. sorghinum* hyphae, RT-qPCR assay is conducted to measure the expression of *CsMYB1*, *CsPME41*, and csi-miR858-3p_L-1 in the first, second, and third leaves of detached tea twigs. The disease lesion area on the inoculated tea leaves gradually increase, compared with that on the control leaves over time (Fig. 8A). RT-qPCR analysis shows that *CsMYB1* expression was downregulated at 12 and 36 hpi in the first leaf and subsequently upregulated at 24 and 48 hpi in the first, second, and third leaves (Fig. 8B-D). In addition, *CsPME41* expression is significantly upregulated at 24, 36, and 48 hpi (Fig. 8B-D). In contrast, csi-miR858-3p_L-1 expression is upregulated at 36 hpi in the first and third leaves, and downregulated at 24 and 48 hpi in the first, second, and third leaves (Fig. 8B-D). Overall, the expression of csi-miR858-3p_L-1, *CsMYB1*, and *CsPME41* exhibited the correlated patterns of change in tea leaves inoculated with *E. sorghinum*.

## Discussion

Tea leaf spot, caused by *E. sorghinum*, severely affects both the yield and quality of tea leaves. Therefore, through high-throughput sequencing of tea leaves inoculated with *E. sorghinum* hyphae, we aim to identify the differentially expressed miRNAs (DEmiRNAs) and DEGs, and investigate the regulatory modes between the DEmiRNAs and DEGs to explore the underlying host–pathogen interaction mechanisms. Compared with the uninoculated samples, we identified 1,068 upregulated DEGs and 467 downregulated DEGs in the inoculated samples. The expression of nine DEmiRNAs was upregulated, while seven DEmiRNAs were significantly downregulated (Huang et al. 2023a, b). Bioinformatic analysis predicted that the csi-miR858-3p_L-1–*CsMYB1* pair is related to host–pathogen interactions. Phylogenetic tree analysis, subcellular localization assay, and self-activation assay confirmed that CsMYB1 is a TF (Fig. 1A, C, D).

We further confirmed the role of CsMYB1 in disease resistance by employing transgenic overexpression of *CsMYB1* mRNA in *N. benthamiana* leaves, transient overexpression of *CsMYB1* mRNA in tea plants, and the transiently silencing of *CsMYB1* mRNA in tea leaves (Fig. 2A-K). Through transient overexpression of *CsMYB1* in tea leaves, combined with transcriptomic and metabolomic assays, as well as a DAP-seq assay, we found that CsMYB1 could upregulate 5,560 DEGs and may directly bind to 24 potential target genes of CsMYB1 (Figs. 3A-F and 4A-D). Furthermore, we identified a new target gene of CsMYB1, *CsPME41* (Fig. 4F-J) and *CsPME41* positively regulated disease resistance in tea plants (Fig. 5A-F). With the aid of degradome sequencing, GUS staining, and Dual-LUC assays, we further confirmed that csi-miR858-3p_L-1 can cleave *CsMYB1* mRNA, suppressing *CsMYB1* mRNA expression (Fig. 6A-D). To investigate whether this effect by csi-miR858-3p_L-1 could reduce disease resistance, we performed *N. benthamiana* transformation with csi-miR858-3p_L-1, along with transient overexpression and transiently silencing of csi-miR858-3p_L-1 in tea plants. Our results indicated that csi-miR858-3p_L-1 negatively regulates the resistance of tea plants to *E. sorghinum* infection (Fig. 7A-I). In an investigation of the regulatory mechanism achieved by CsMYB1, we observed a dynamic interaction between *CsMYB1*, *CsPME41*, and csi-miR858-3p_L-1 in terms of altered mRNA abundance following tea leaf infection by *E. sorghinum* hyphae (Fig. 8A-D).

This study is the first to report the disease resistance function of CsMYB1 in tea leaves. Previously, it had been reported that CsMYB1 is involved in regulating trichome formation and the biosynthesis of gallic acid-conjugated catechins in tea plants (Li et al., 2022). In other plant species, studies on MYB1 have mostly focused on plant growth, development, and responses to abiotic stress. For example, GhMYB1a regulated the accumulation of flavonols and anthocyanins in *Gerbera* × *hybrida* (Zhong et al. 2020), while MYB1 also coordinated abscisic acid biosynthesis and signaling during salt stress in *Arabidopsis* (Wang et al. 2015).

This study also marks the first report of CsMYB1 actively regulating the host plant's defense response to fungal disease by activating *CsPME41*, while it is also the first time that *CsPME41* gene has been identified in tea plants. PME is a ubiquitous cell wall enzyme involved in various developmental processes and responses to both biotic and abiotic stresses. For example, low-temperature and 24-epibrassinolide treatments induced the expression of *AtPME41* mRNA, with brassinosteroids regulating PME activity under cold stress by modulating the expression of *AtPME41* mRNA (Qu et al. 2011). PME played a critical role in maintaining cell wall integrity and is essential in plant–fungal interactions (Lionetti et al. 2007, 2012). In *G. hirsutum*, GhPME2/GhPME31 participated in defense against the fungal pathogen *Verticillium dahliae* (Liu et al. 2018).

In addition, this study firstly discovers a novel miRNA in tea plants, namely csi-miR858-3p_L-1, and demonstrates that it negatively regulates the tea plant’s disease resistance mechanism by targeting *CsMYB1*. In *Arabidopsis*, overexpression of MIR858 increased the plant's sensitivity to fungal pathogens (Camargo-Ramírez et al. 2018), a finding which is consistent with the result from our study. In apple, miR858 targeted and repressed the expression of *MdMYB9*, *MdMYB11*, and *MdMYB12* to control the biosynthesis of proanthocyanidins in apple peel (Zhang et al. 2022).

Although this study is the first to reveal the molecular mechanism by which the csi-miR858-3p_L-1–*CsMYB1*–*CsPME41* module regulates disease resistance in tea plants, several limitations exist in the interpretation of the data. First, previous studies shown that PME enhances disease resistance by regulating cell wall modifications and pathogen resistance in various plant species (Lionetti et al. 2012); nevertheless, its specific mechanism of action in tea plants still requires further investigation. Second, the experiments were conducted using genetic transformation in *N. benthamiana*. Due to the challenges associated with genetic transformation in tea plants, we have not yet performed transformation of *CsMYB1*, *CsPME41*, and csi-miR858-3p_L-1 genes in tea plants to confirm the underlying mechanisms on disease resistance. Future research should focus on analyzing more complex regulatory networks and exploring the detailed mechanisms through which TFs interact with the promoters of *CsMYB1*, *CsPME41*, and csi-miR858-3p_L-1 genes, to uncover the upstream or downstream regulatory mechanisms of miRNAs. A more comprehensive understanding of the role of CsPME41, through multi-omics analysis and integration of experimental data, will aid in clarifying its role in regulating pectin methylesterification and maintaining cell wall integrity, thus providing stronger theoretical support for breeding disease-resistant tea plants.

## Materials and methods

### Plant materials and pathogens

Three-year-old tea plant (*C. sinensis* cv. Fuding-dabaicha), were cultivated under controlled conditions (25/20°C day/night temperature regime, 14-h light/10-h dark photoperiod, 70–80% relative humidity) in the greenhouse at Guizhou University, Guiyang City, Guizhou Province, China (E26°25', N106°40'), were used as the experimental plant material. *N. benthamiana* was cultivated in a greenhouse under long-day (16-h light/8-h dark) conditions at 24 °C with 70–80% relative humidity. The pathogens *E. sorghinum* strain CGMCC3.20150 and *B. cinerea* strain CGMCC3.20932 were cultured on potato dextrose agar (PDA), and virulence assay were performed following the methods used in our research laboratory (Guo et al. 2025).

### Total RNA extraction and RT-qPCR

Total RNA was extracted using the TransZol Up Plus RNA Kit (TransGen, Beijing, China). mRNA and miRNA were reverse transcribed into cDNA using Hifair AdvanceFast 1 st Strand cDNA Synthesis SuperMix for RT-qPCR (DNA digester plus) (Yeasen, Shanghai, China) and Hifair III 1 st Strand cDNA Synthesis Kit (gDNA digester plus) (Yeasen, Shanghai, China), respectively. All primers were designed using the NCBI Primer-BLAST tool (https://www.ncbi.nlm.nih.gov/tools/primer-blast/) and synthesized by Sangon Biotech (Shanghai) Co., Ltd., Shanghai, China. RT-qPCR was performed using Hieff UNICON Advanced qPCR SYBR Master Mix (Yeasen, Shanghai, China) on the Applied Biosystems QuantStudio 6 Flex Real-Time PCR System (Thermo Fisher Scientific, Waltham, MA, USA). For mRNA and miRNA expression analysis, *CsGAPDH* and Csn-miR222, respectively, were used as internal reference genes in tea plants. The relative expression levels were calculated using the 2^−ΔΔ*C*t^ method (Livak and Schmittgen 2001). The primers used are listed in Supplemental Table S6.

### Bioinformatic analyses of CsMYB1

The protein sequence information for CsMYB1 was obtained from the Tea Plant Information Archive (TPIA; http://tpia.teaplant.org). The relevant protein sequences for *Arabidopsis* were retrieved from The Arabidopsis Information Resource (TAIR; http://www.arabidopsis.org/). Multiple sequence alignment was performed using ClustalW software (https://www.genome.jp/tools-bin/clustalw). A phylogenetic tree was constructed using the Neighbor-Joining method in MEGA 11, with 1,000 bootstrap replicates. Protein sequences were compared, and domain analysis was conducted using the GeneDoc software (version 2.7, http://nrbsc.org/gfx/genedoc/index.html).

### Subcellular localization assay

The coding sequence (CDS) of *CsMYB1* with free terminator was ligated to the pCAMBIA2300 vector via *Bam*HI and *SaI*I to produce the fusion vector 35SCaMV::*CsMYB1-GFP*. The pCAMBIA2300-*CsMYB1*-*GFP* construct and pBI121-*NLS*-*mCherry* nuclear marker construct were transformed into *A. tumefaciens* strain GV3101 (pSoup-p19). When the bacterial culture reached an OD_600_ = 1.0, the pCAMBIA2300-*CsMYB1*-*GFP* and pBI121-*NLS*-*mCherry* constructs were mixed at a 1:1 ratio and infiltrated into leaves of *N. benthamiana*, with the pCAMBIA2300 empty vector being used as the negative control. After 2 days, the leaves were imaged using a Zeiss confocal microscope LSM 900 (Carl Zeiss, Oberkochen, Germany). The primers used are listed in Supplemental Table S6.

### Transactivation activity assay

The CDS of *CsMYB1* was cloned into the pGBKT7 vector. Subsequently, the pGBKT7-*CsMYB1* and pGBKT7 vectors were transformed into the yeast strain AH109. Following positive PCR detection, individual colonies of positive clones were selected and grown on SD media (SD/− Trp, SD/− Trp − His, and SD/− Trp − His/X-*α*-gal, SD/− Trp − His − Ade, and SD/− Trp − His − Ade/X-*α*-gal) in the dark at a constant temperature of 30 °C for 3 days. The primers used are listed in Supplemental Table S6.

### Genetic transformation of N. benthamiana

The generation of transgenic *N. benthamiana* was performed as described previously, with minor modifications (Khare et al. 2010). The CDS of *CsMYB1* without free terminator and the precursor sequence of csi-miR858-3p_L-1 was inserted into the pBI121 vector. Explants were prepared from excised cotyledons obtained from 2-month-old *N. benthamiana* seedlings on Murashige and Skoog (MS) medium using the leaf disk method and then inoculated by infiltration with *A. tumefaciens* strain GV3101 containing the pBI121-*CsMYB1* and pBI121-csi-miR858-3p_L-1 vectors. The positive plants were selected on MS medium containing 50 mg/L kanamycin. DNA was extracted from *N. benthamiana* leaves, and PCR was used to verify the transgenic plants using specific primers, and the transgenic plants were inoculated with *B. cinerea*; WT *N. benthamiana* was used as the control in the resistance assay. The primers used are listed in Supplemental Table S6.

### Transient overexpression in tea plants

The CDS region of *CsMYB1* and *CsPME41*, and the precursor sequence of csi-miR858-3p_L-1 was inserted into the pBI121 vector and transferred to *A. tumefaciens* strain GV3101, with the pBI121 empty vector being used as a control. Subsequently, *A. tumefaciens* strain GV3101 containing pBI121-*CsMYB1*, pBI121-*CsPME41*, pBI121-csi-miR858-3p_L-1, or the pBI121 empty vector were injected into the abaxial surface of tea leaves. After 48 h incubation, the injected leaves were sampled for RT-qPCR assay. At 48 hpi with *E. sorghinum* hyphae in injected leaves, the lesion areas on tea leaves were measured. The primers used are listed in Supplemental Table S6.

### AsODN assay in detached tea twigs

To investigate whether *CsMYB1* mRNA, *CsPME41* mRNA, and csi-miR858-3p_L-1 are involved in tea plant resistance to pathogens, AsODN assay was used to transiently silence *CsMYB1* mRNA, *CsPME41* mRNA, and csi-miR858-3p_L-1 in tea leaves and test the resistance of tea leaves to *E. sorghinum*. The Soligo online tool (https://sfold.wadsworth.org/cgi-bin/soligo.pl) was used to select the appropriate AsODNs sequences for *CsMYB1* mRNA, *CsPME41* mRNA, and csi-miR858-3p_L-1. The AsODN procedure was performed as described previously (Li et al. 2022b). In brief, healthy one-bud, two-leaf detached tea shoots were placed into 1.5 mL microcentrifuge tubes each containing 20 μM AsODN-*CsMYB1*, AsODN-*CsPME41*, and AsODN-csi-miR858-3p_L-1 for 48 h. The first leaf of each shoot was collected for RT-qPCR assay of *CsMYB1* mRNA, *CsPME41* mRNA, csi-miR858-3p_L-1; the second leaf of each shoot was inoculated with two PDA plugs containing *E. sorghinum* hyphae, and the lesions areas were measured at 48 hpi (Guo et al. 2025). The primers used are listed in Supplemental Table S6.

### Biomass analysis of E. sorghinum using qPCR

After transient overexpression and transient silencing of *CsMYB1* in tea leaves followed by inoculation with *E. sorghinum*, lesion areas were collected. Genomic DNA was extracted from tea leaves using the CTAB method. qPCR was performed using Hieff UNICON Advanced qPCR SYBR Master Mix (Yeasen, Shanghai, China) on an Applied Biosystems QuantStudio 6 Flex Real-Time PCR System (Thermo Fisher Scientific). Each reaction contained 100 ng DNA and *E. sorghinum*-specific primers. *CsGAPDH* was used as an endogenous reference to normalize the samples. Genes expression levels were quantified using the 2^−ΔΔ*C*t^ method (Livak and Schmittgen 2001). The primers used are listed in Supplemental Table S6.

### Transcriptomic and metabolomic assays

*A. tumefaciens* strain GV3101 containing the pBI121-*CsMYB1* vector was injected into the vascular bundles of tea leaves. After 2 days, the tissues were collected from the transiently overexpressed tea leaves. Sequencing libraries were constructed according to the manufacturer’s instructions and sequenced to generate 150-bp paired-end reads on an Illumina NovaSeq 6000 platform (LC Science, Hangzhou, China). The high-quality clean data were annotated using the reference genome of *C. sinensis* (ShuchazaoV1) (https://tpia.teaplants.cn/download.html) (Qi et al. 2025). Finally, selected DEGs of interest were validated by RT-qPCR assay. The primers used are listed in Supplemental Table S6.

Metabolomic assay was conducted according to previously established protocols, with minor modifications (Li et al. 2025). Metabolites were extracted from the tea leaf tissues, using 50% (v/v) methanol, and metabolites eluted from the ACQUITY UPLC HSS T3 column (Waters, Milford, MA, USA) were detected using a high-resolution tandem mass spectrometer, Q-Exactive (Thermo Fisher Scientific, Waltham, MA, USA). Peak extraction and quality control were performed using XCMS software (https://github.com/sneumann/xcms) (Smith et al. 2006). Metabolite identification was carried out using the metaX software (http://metax.genomics.cn) (Wen et al. 2017). Finally, bioinformatic analysis of the metabolites was conducted using the online KEGG (https://www.kegg.jp/) and HMDB databases (https://hmdb.ca) (Kanehisa et al. 2000; Wishart et al. 2022).

The correlation analysis of transcriptomic and metabolomic data was performed using Pearson’s correlation coefficient (r) (Ma et al. 2015). The calculations were conducted using the ‘corr’ package in R, with the selection criteria set to |r|> 0.9 and *P* < 0.01. Finally, the association network between the two datasets was visualized using Cytoscape (version 3.7.2, Cytoscape Consortium, San Diego, CA, USA).

### DAP-seq assay

DAP-seq assay was performed according to a previously published method (Orduña et al. 2022). Briefly, total DNA was extracted from leaves of the tea plant, purified, fragmented, and ligated to short DNA-sequencing adapters to generate the DAP-seq library. The CDS of *CsMYB1* was inserted into a pET-30a-HaloTag vector and then expressed in TnT SP6 High-Yield Germ Master Mix (Promega Biotech, Beijing, China). The fusion protein was purified using the MANAGE HaloTag Beads (Promega Biotech, Beijing, China). After the fusion protein was bound to the DNA, the unbound DNA was washed away. The bound DNA was then used to generate sequencing libraries, which were subsequently analyzed on an Illumina HiSeq 6000 platform (LC Science, Hangzhou, China). Enriched peaks were mapped to the TPIA database to identify target genes of CsMYB1 and to recognize motifs. The target genes were annotated using GO enrichment analysis (version 0.9.9, https://github.com/tanghaibao/GOatools) and KEGG enrichment analysis (version 3.0, https://www.kegg.jp/). A scatter plot for the DEGs was generated. Fisher's exact probability test was used for statistical analysis. Conserved motifs in the peaks were identified using MEME-ChIP software (version 5.0.5, https://meme-suite.org/meme/tools/meme-chip).

### EMSA

EMSA was performed as previously described (Zhao et al. 2025), with minor modifications. The CDS of *CsMYB1* was cloned into the pET-30a-HaloTag expression vector, and the recombinant His-CsMYB1 fusion protein was induced and expressed in BL21(DE3) competent cells. The protein was purified using Ni-IDA-Sepharose Cl-6B affinity chromatography resin (Novagen, MilliporeSigma, USA). Subsequently, the purified recombinant protein was incubated with a biotin-labeled wild probe, a corresponding unlabeled cold competitor probe, and a biotin-labeled mutant competitor probe using a chemiluminescent EMSA kit (Beyotime, Shanghai, China). Finally, the results were detected and imaged using a chemiluminescence imaging system (Qinxiang, Shanghai, China). The probes were synthesized by Sangon Biotech (Shanghai) Co., Ltd., Shanghai, China, and are listed in Supplemental Table S6.

### Y1H assay

Y1H assay was conducted as described previously (Huai et al. 2022). Briefly, the CDS of *CsMYB1* was cloned into the pGADT7 vector. The promoter of *CsPME41*, containing the *CsMYB1 cis*-regulatory element, was cloned into the pAbAi vector. The plasmid was linearized using *Bst*BI and transformed into the yeast strain Y1H Gold (Pyeast, Wuhan, China), which was plated onto SD/− Ura selective medium (Pyeast, Wuhan, China). The pGADT7-*CsMYB1* vector was transformed into yeast prepared by pAbAi-pro-*CsPME41* and plated on SD/− Leu selective medium containing the selected AbA concentration (0, 50, or 150 ng/mL). A pGADT7 empty vector was used as a negative control, and pAbAi-*p53* was used as a positive control. The primers used are listed in Supplemental Table S6.

### Dual-LUC assay

Dual-LUC assay was performed as described previously (Zhang et al. 2024), with minor modifications. To validate the interaction between CsMYB1 and its target gene *CsPME41*, the promoter of *CsPME41* gene was cloned into the pGreenII 0800-*LUC* vector as a reporter gene. The CDS of *CsMYB1* gene was cloned into the pGreenII 62-SK vector as an effector. The constructed plasmids containing both the reporter and effector were then transformed into *A. tumefaciens* strain GV3101 (pSoup) and co-infiltrated into *N. benthamiana* leaves. Different regions of the same *N. benthamiana* leaf were injected with pGreen II 62-SK-*CsMYB1* + pGreen II 0800-*LUC*-pro-*CsPME41*, pGreen II 62-SK empty vector + pGreen II 0800-*LUC*-pro-*CsPME41*, and pGreen II 62-SK-*CsMYB1* + pGreen II 0800-*LUC* empty vector, and incubated for 2 days. D-Luciferin potassium salt (Biyuntian, Shanghai, China) was dissolved in sterile phosphate-buffered saline (free of Mg^2+^ and Ca^2+^) (pH 7.4, 0.01 M phosphate) to prepare a 15 mg/mL solution. The solution was filtered through a 0.2-μm pore-size filter and then diluted with sterile water to 1:200 to achieve a final concentration of 150 μg/mL. After incubation at 37 °C for 5 min, luminescence on *N. benthamiana* leaves was observed and quantified using a chemiluminescence imaging system (Qinxiang, Shanghai, China). The activities of *Firefly* luciferase (LUC) and *Renilla* luciferase (REN) were measured using a Dual-Luciferase Reporter Gene Assay Kit (Yeasen, Shanghai, China) on a Feyond-A300 multi-functional microplate reader (Allsheng, Hangzhou, China), with the LUC/REN ratio calculated for each sample. To validate the interaction between csi-miR858-3p_L-1 and its target gene *CsMYB1*, we cloned the *CsMYB1* as a reporter gene into the pGreenII 0800-*LUC* vector. The precursor of csi-miR858-3p_L-1 was cloned as an effector into the pGreenII 62-SK vector, following the same methodology described above. The primers used are listed in Supplemental Table S6.

### Degradome sequencing assay

Degradome sequencing assay was employed to investigate miRNA target genes. The experimental procedure was primarily based on a previous study (Lin et al. 2016), with minor modifications. Specifically, total RNA was extracted from uninoculated leaves (control) and inoculated leaves (treatment), and, as a result, two independent degradome libraries were constructed. Potential miRNA target genes were identified using the ACGT101-DEG tool (LC Sciences, http://www.lcsciences.com/) and the CleaveLand 3.0 software package (https://sites.psu.edu/axtell/software/cleaveland3/).

### Histochemical GUS staining assay

The CDS of *CsMYB1* gene and the csi-miR858-3p_L-1 precursor sequence was independently inserted into the pBI121 vector and transferred into receptor cells of *A. tumefaciens* strain GV3101. Two days after transformation, *N. benthamiana* leaves were stained with GUS as previously described (Jefferson et al. 1987). The pBI121-*GUS* vector was used as a negative control. The primers used are listed in Supplemental Table S6.

### Changes in CsMYB1 mRNA, CsPME41 mRNA, and csi-miR858-3p_L-1 abundance in tea leaves inoculated with E. sorghinum

The first, second, and third leaves on detached tea twigs were inoculated with *E. sorghinum* mycelium from PDA cultures. Lesions on tea leaves were observed and measured at 12, 24, 36, and 48 hpi. Tissue samples from the lesion margins, where diseased and healthy tissues intersect, were harvested using a sterile hole punch for RT-qPCR assay, with the sample at 0 hpi being used as the control (Huang et al. 2023a, b). Furthermore, the expression of csi-miR858-3p_L-1 was detected using a stem-loop RT-qPCR assay. Total RNA was reverse transcribed using a miRNA-specific stem-loop RT primer. This primer hybridizes to the 3′ end of the mature miRNA, forming a stem-loop structure that enhances specificity and facilitates cDNA synthesis. Subsequently, PCR was performed. The primers used are listed in Supplemental Table S6.

### Statistical analysis

Statistical analyses were performed using Prism 9.0 (GraphPad, San Diego, CA, USA). Data were presented as mean ± standard deviation. For multiple comparisons (of more than two samples), *P*-values were calculated using one-way ANOVA and the Tukey multiple comparison test. The Student’s *t*-test was used to compare two samples.

## Supplementary Information

Supplementary Material 1. Fig. S1 Volcano map of differentially expressed genes.

Supplementary Material 2. Fig. S2 Volcano map of differentially abundant metabolites.

Supplementary Material 3. Fig. S3 The average abundance of reads near CsMYB1-binding regions within the 10-kb region upstream and downstream of the transcription start site (TSS) for CsMYB1-binding region.

Supplementary Material 4. Fig. S4 Distribution of peak length by DAP-seq.

Supplementary Material 5. Table S1 Summary of differentially expressed genes between CsMYB1-OE vs. pBI121 control tea leaves. Table S2 Summary of differential abundant metabolites between CsMYB1-OE vs. pBI121 control tea leaves. Table S3 Interrelationships between seven stress-related mRNAs and metabolites in CsMYB1-OE and pBI121 control tea leaves. Table S4 RT-qPCR validation of 10 differentially expressed genes. Table S5 DNA affinity purification sequencing data. Table S6 The primers, oligonucleotide sequence, and probes used in this study.

## Data Availability

The raw RNA-seq and small RNA-seq data generated in this study have been deposited in the NCBI SRA under the following BioProject accession numbers: PRJNA799860 (mRNA-seq from *C. sinensis* leaves infected with *E. sorghinum*), PRJNA824005 (small RNA-seq from *C. sinensis* leaves infected with *E. sorghinum*), and PRJNA1248450 (mRNA-seq from *C. sinensis* leaves transiently overexpressing *CsMYB1*). Sequence data from this article can be found in the Tea Plant Information Archive (https://tpia.teaplants.cn/index.html) as follows: *CsMYB1* (TEA008298), *CsPME41* (TEA023780), *CsRS1* (TEA003419), *CsPK* (TEA004578), *CsCSP41b* (TEA006794), *CsRAP2-12* (TEA013180), *CsCASR* (TEA014099), *CsSRSF* (TEA017339), *CsWRKY* (TEA020191), *CsLAC7* (TEA020236), *CsPAE* (TEA022428), *CsPOD* (TEA022953). All data generated and analyzed during this study are included in this published article and its supplementary information files (Table S1-S5). Moreover, all other data are available from the corresponding author upon reasonable request.

## Abbreviations

AsODN: Antisense oligonucleotide
DAMs: Differentially abundant metabolites
DAP-seq: DNA affinity purification sequencing
DEGs: Differentially expressed genes
Dual-LUC: Dual-luciferase
EMSA: Electrophoretic mobility shift assay
GUS: *β*-Glucuronidase
miRNAs: MicroRNAs
sODN: Sense oligonucleotide
PME41: Pectinesterase/pectinesterase inhibitor 41
TFs: Transcription factors
Y1H: Yeast one-hybrid
