# Supplementary figures and images for: A miR858 variant negatively regulates resistance to tea leaf spot through targeting the CsMYB1–CsPME41 module
Source: Mol Hortic. 2026 Jul 7;6:51. doi: 10.1186/s43897-026-00238-7 (PMC13339706; doi:10.1186/s43897-026-00238-7)

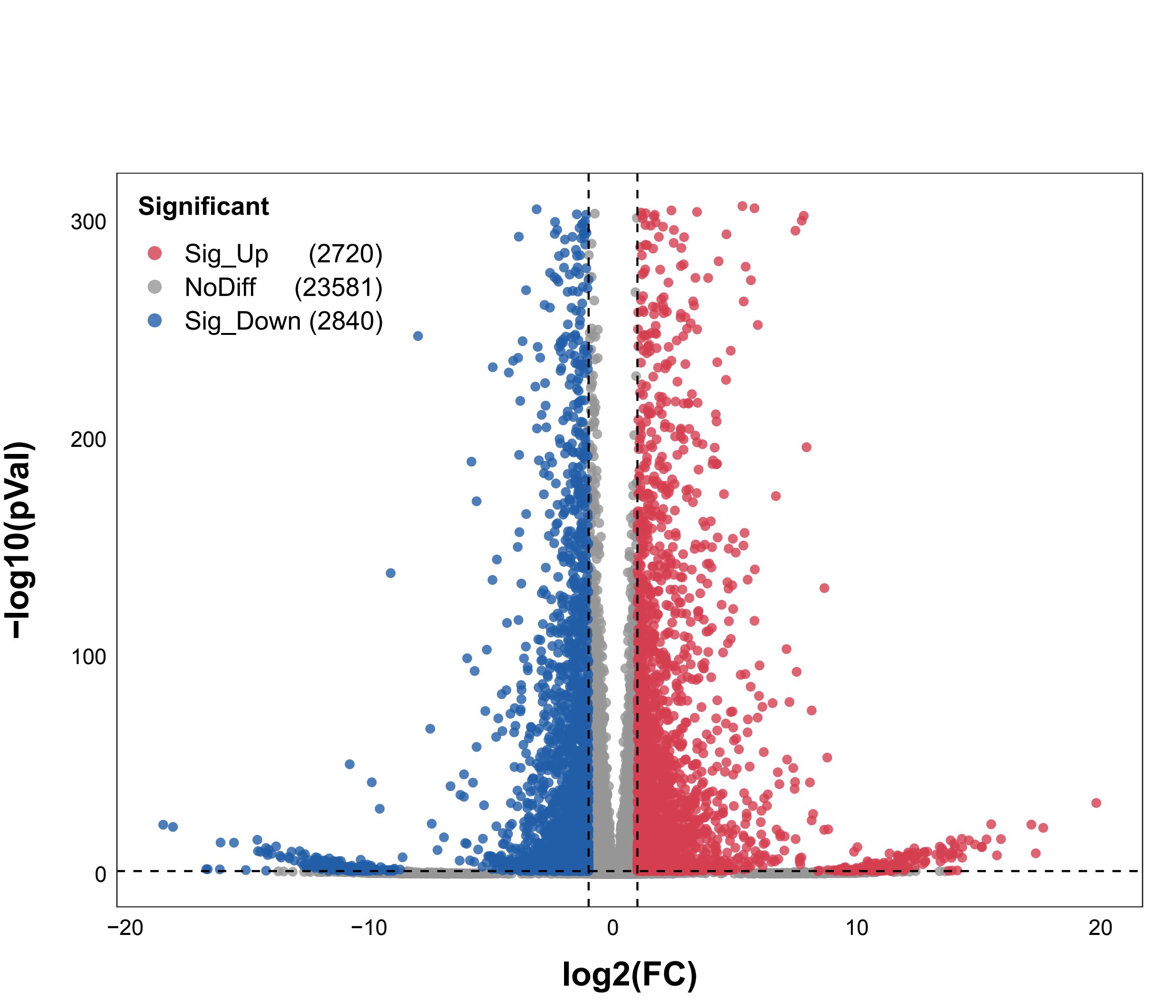

Supplement: Supplementary file 1 — Supplementary Material 1. Fig. S1 Volcano map of differentially expressed genes. [file 43897_2026_238_MOESM1_ESM.tiff]

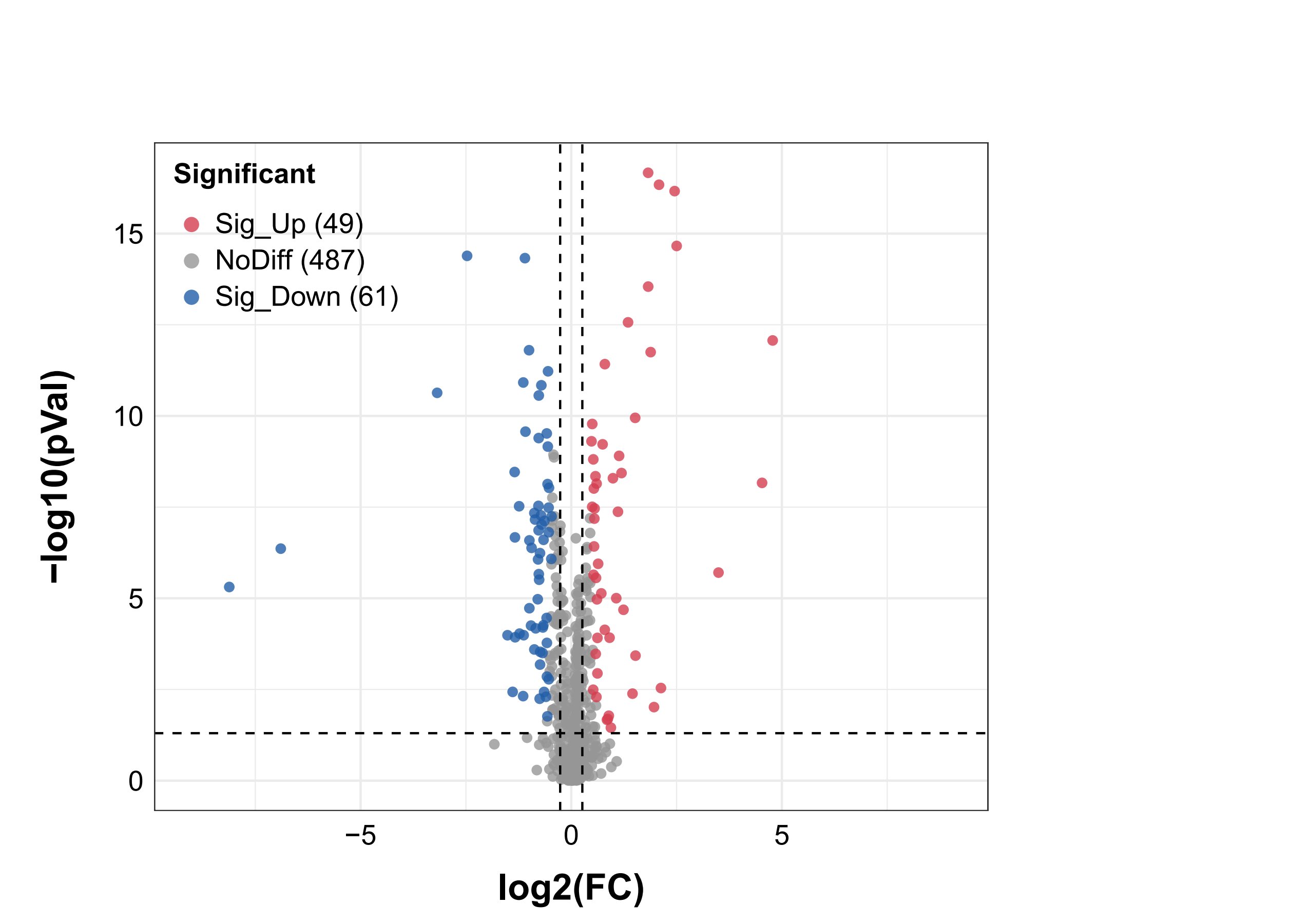

Supplement: Supplementary file 2 — Supplementary Material 2. Fig. S2 Volcano map of differentially abundant metabolites. [file 43897_2026_238_MOESM2_ESM.tiff]

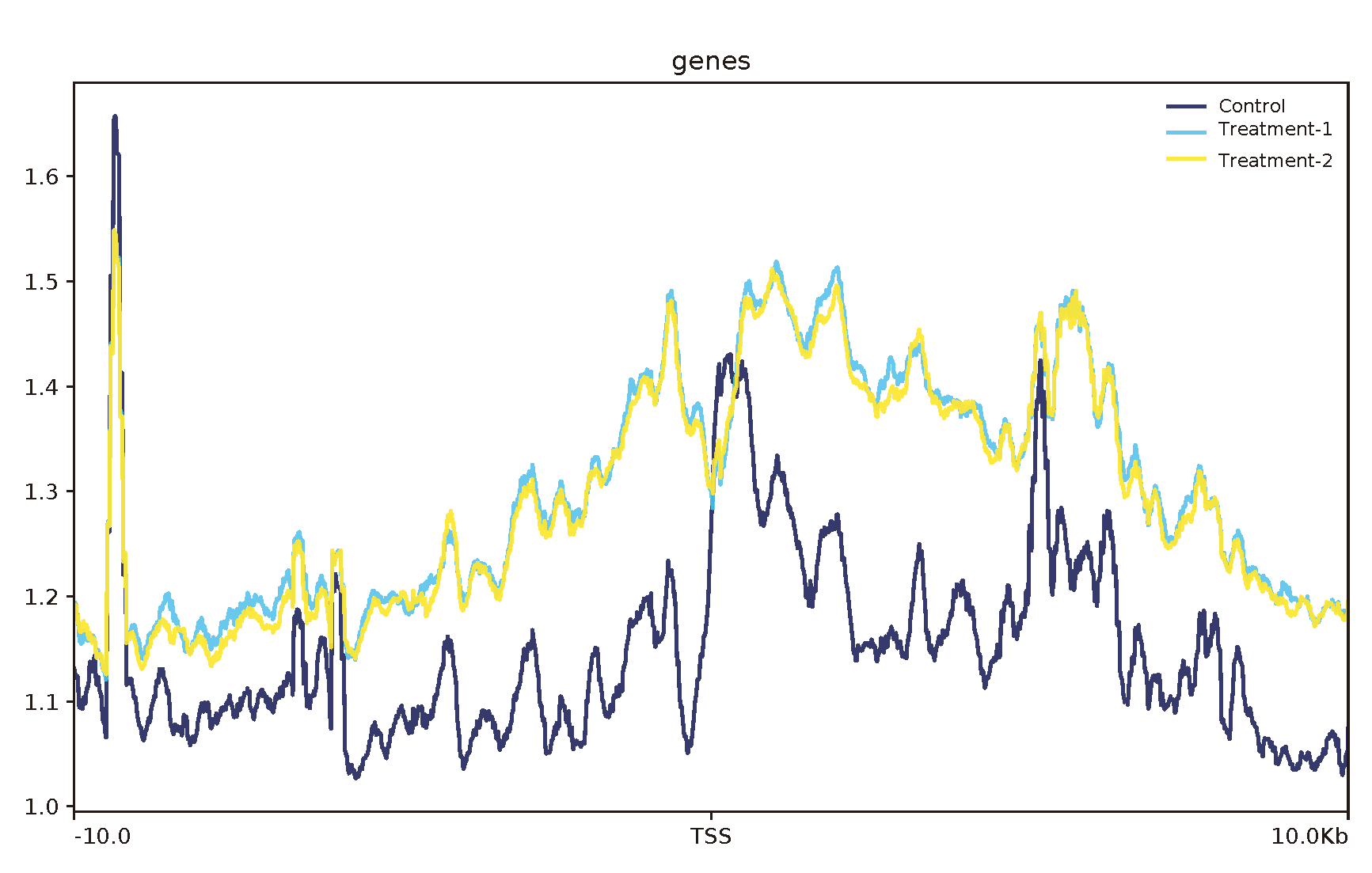

Supplement: Supplementary file 3 — Supplementary Material 3. Fig. S3 The average abundance of reads near CsMYB1-binding regions within the 10-kb region upstream and downstream of the transcription start site (TSS) for CsMYB1-binding region. [file 43897_2026_238_MOESM3_ESM.tiff]

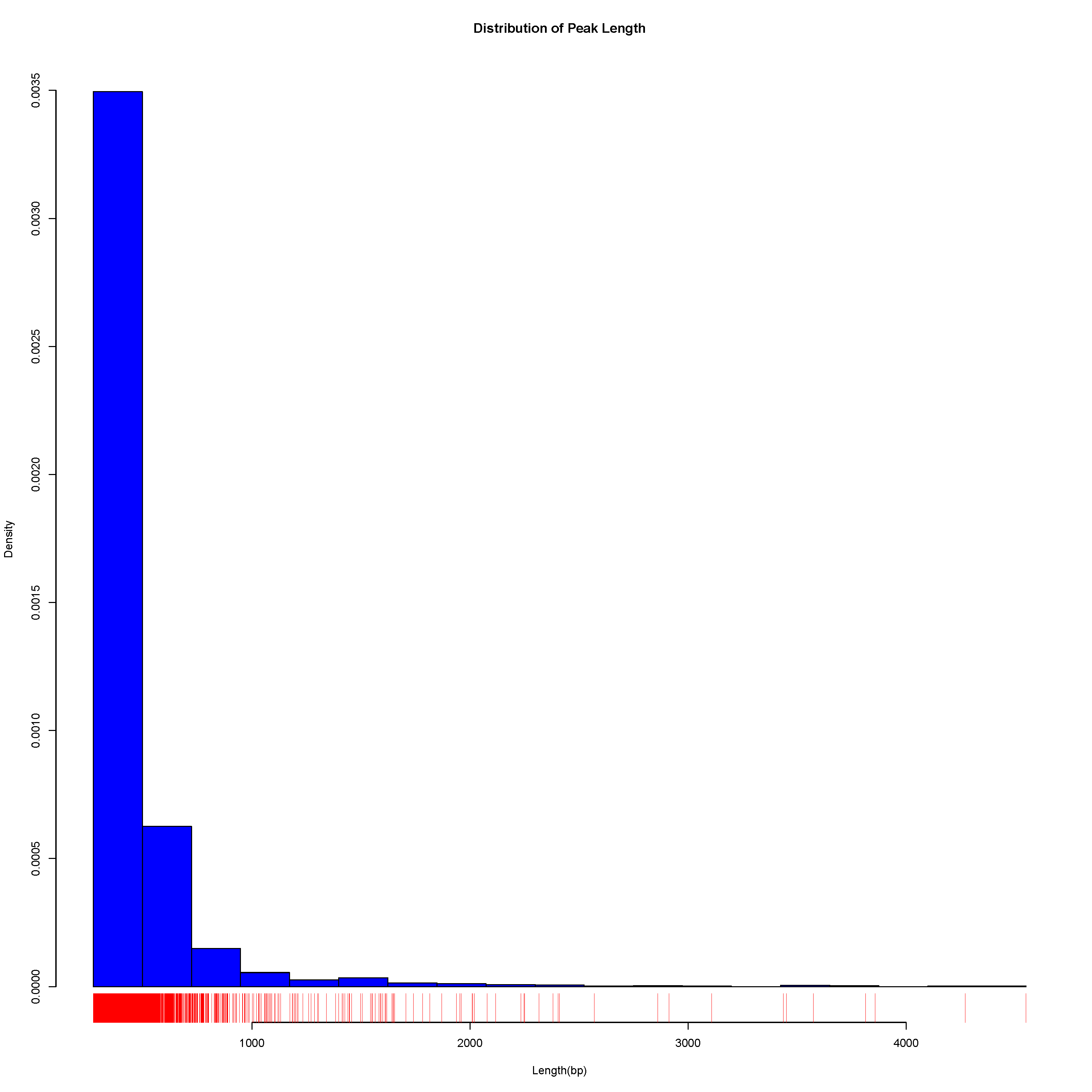

Supplement: Supplementary file 4 — Supplementary Material 4. Fig. S4 Distribution of peak length by DAP-seq. [file 43897_2026_238_MOESM4_ESM.tiff]
